# Long-term ambient air pollution exposure and renal function and biomarkers of renal disease

**DOI:** 10.1186/s12940-024-01108-9

**Published:** 2024-08-09

**Authors:** Karl Kilbo Edlund, Yiyi Xu, Eva M. Andersson, Anders Christensson, Mats Dehlin, Helena Forsblad-d’Elia, Florencia Harari, Stefan Ljunggren, Peter Molnár, Anna Oudin, Magnus Svartengren, Petter Ljungman, Leo Stockfelt

**Affiliations:** 1 Occupational and Environmental Medicine, School of Public Health and Community Medicine, Institute of Medicine, Sahlgrenska Academy, University of Gothenburg, Box 414, 405 30 Gothenburg, Sweden; 2 Occupational and Environmental Medicine, Sahlgrenska University Hospital, Gothenburg, Sweden; 3 Department of Nephrology, Skåne University Hospital, Lund University, Malmö, Sweden; 4 Department of Rheumatology and Inflammation Research, Institute of Medicine, Sahlgrenska Academy, University of Gothenburg, Göteborg, Sweden; 5 Occupational and Environmental Medicine Center in Linköping, and, Department of Health, Medicine and Caring Sciences, Linköping University, Linköping, Sweden; 6 Division of Occupational and Environmental MedicineDepartment of Laboratory MedicineFaculty of Medicine, Lund University, Lund, Sweden; 7 Division for Sustainable Health, Department of Public Health and Clinical Medicine, Faculty of Medicine, Umeå University, Umeå, Sweden; 8 Department of Medical Sciences, Faculty of Medicine, Uppsala University, Uppsala, Sweden; 9 Institute of Environmental Medicine, Karolinska Institute, Stockholm, Sweden; 10 Department of Cardiology, Danderyd Hospital, Stockholm, Sweden

**Keywords:** Air pollution, Kidney disease, eGFR, Biomarkers, Matrix metalloproteinases

## Abstract

**Background:**

Despite accumulating evidence of an association between air pollution and renal disease, studies on the association between long-term exposure to air pollution and renal function are still contradictory. This study aimed to investigate this association in a large population with relatively low exposure and with improved estimation of renal function as well as renal injury biomarkers.

**Methods:**

We performed a cross-sectional analysis in the middle-aged general population participating in the Swedish CardioPulmonary bioImaging Study (SCAPIS; *n* = 30 154). Individual 10-year exposure to total and locally emitted fine particulate matter (PM_2.5_), inhalable particulate matter (PM_10_), and nitrogen oxides (NO_x_) were modelled using high-resolution dispersion models. Linear regression models were used to estimate associations between exposures and estimated glomerular filtration rate (eGFR, combined creatinine and cystatin C) and serum levels of renal injury biomarkers (KIM-1, MCP-1, IL-6, IL-18, MMP-2, MMP-7, MMP-9, FGF-23, and uric acid), with consideration of potential confounders.

**Results:**

Median long-term PM_2.5_ exposure was 6.2 µg/m^3^. Almost all participants had a normal renal function and median eGFR was 99.2 mL/min/1.73 m^2^. PM_2.5_ exposure was associated with 1.3% (95% CI 0.6, 2.0) higher eGFR per 2.03 µg/m^3^ (interquartile range, IQR). PM_2.5_ exposure was also associated with elevated serum matrix metalloproteinase 2 (MMP-2) concentration, with 7.2% (95% CI 1.9, 12.8) higher MMP-2 per 2.03 µg/m^3^. There was a tendency towards an association between PM_10_ and higher levels of uric acid, but no associations were found with the other biomarkers. Associations with other air pollutants were null or inconsistent.

**Conclusion:**

In this large general population sample at low exposure levels, we found a surprising association between PM_2.5_ exposure and a higher renal filtration. It seems unlikely that particle function would improve renal function. However, increased filtration is an early sign of renal injury and may be related to the relatively healthy population at comparatively low exposure levels. Furthermore, PM_2.5_ exposure was associated with higher serum concentrations of MMP-2, an early indicator of renal and cardiovascular pathology.

**Supplementary Information:**

The online version contains supplementary material available at 10.1186/s12940-024-01108-9.

## Background

Chronic kidney disease (CKD) is estimated to directly cause 1.2 million premature deaths annually and to indirectly cause a further 1.4 million premature deaths through cardiovascular diseases attributable to impaired kidney function [7]. A number of recent epidemiological studies have shown an association between exposure to air pollution and CKD, including a recent study at low exposure levels in southern Sweden [36, 62, 83]. The epidemiological evidence has been corroborated by experimental studies that have shown renal vascular dysfunction, renal fibrosis, glomerular and tubular injury, and markers of impaired filtration capacity following both acute and sub-chronic exposure to high particle levels [1, 4, 10, 30, 49, 50, 78, 84]. However, previous epidemiological studies of the biological intermediaries of chronic kidney disease, i.e., impaired renal function and albuminuria, remain inconclusive.

Exposure to air pollution may affect the kidneys through both direct and indirect routes. Inhaled fine particles can translocate through the bloodstream to the kidney and either deposit in the renal parenchyma or be excreted through the urine [3, 46, 61, 66], which indicates that they are able to directly exert a nephrotoxic effect. Synergism between the renal and cardiovascular effects of air pollution is also plausible, as renal diseases share important biological pathways with cardiovascular disease, such as systemic inflammation, sympathetic nervous system overactivity, renin–angiotensin–aldosterone system (RAAS) activation, volume overload, and renal and cardiac remodelling [67, 77, 80]. Atherosclerotic disease is overrepresented among patients with CKD and may contribute to the pathology already at early stages in the renal disease progression and, conversely, it is also well established that renal dysfunction incurs a higher risk of cardiovascular disease progression, independent of traditional cardiovascular risk factors [11, 21, 22]. It is therefore possible that kidney injury mediates part of the cardiovascular effects of air pollution exposure.

The glomerular filtration rate (GFR) is the primary metric for renal filtration capacity and is generally interpreted as an overall proxy for kidney function. Most often, GFR is estimated (eGFR) from plasma creatinine concentrations. Cystatin C has improved the validity of GFR estimations and the combination of creatinine and cystatin C provides the most accurate estimates [25, 72]. CKD is defined as GFR < 60 mL/min/1.73 m^3^ or > 60 mL/min/1.73 m^3^ in combination with albuminuria. However, GFR decreases only once the renal reserve has been depleted and clinically significant CKD manifests only after substantial decline. Other biomarkers have been proposed for detection of subclinical kidney injury before renal filtration is affected and for better prediction of CKD progression [86]. Among these are serum uric acid [5], kidney injury molecule 1 (KIM-1), monocyte chemoattractant protein 1 (MCP-1), interleukins 6 and 18 (IL-6, IL-18), matrix metalloproteinases 2, 7, and 9 (MMP-2, MMP-7, MMP-9), and fibroblast growth factor 23 (FGF-23) [12, 54, 77, 86]. While several studies have documented the ability of serum biomarkers to predict CKD progression in humans [20, 24, 28, 69], associations between air pollution exposure and these biomarkers have so far received limited scientific attention.

The objectives of this study were to investigate the associations between residential air pollution exposure and eGFR as well as serum levels of novel biomarkers of subclinical kidney injury, in the large-scale population-based Swedish CArdiopulmonary bioImage Study (SCAPIS). Our hypotheses were that higher exposure to ambient air pollution would be associated with a lower eGFR and higher levels of biomarkers of renal inflammation, dysfunction, and injury.

## Method

We performed a cross-sectional study of the association between air pollution exposure and renal function and renal injury in the Swedish CArdioPulmonary bioImaging Study (SCAPIS). SCAPIS is a multi-centre, population-based cohort study in six Swedish cities, which between 2013 and 2018 recruited 30 154 men and women aged 50–64. The overall participation rate was 50.3% [6]. All participants were extensively examined, including blood sampling and a questionnaire collecting detailed information on health, family history, medication, and lifestyle. Both SCAPIS and the current study have received ethical approval (from the Regional Ethics Board of Umeå, Dnr 2010/228–31, and from the Swedish Ethical Review Authority, Dnr 2022–00789-02, respectively). All participants provided written informed consent.

### Exposure and co-exposure assessment

Ambient concentrations of total and locally emitted fine particulate matter (PM_2.5_), inhalable particulate matter (PM_10_), and nitrogen oxides (NO_x_) were modelled at a dynamic resolution up to 50 × 50 m^2^, dependent on proximity to emission sources, using methods described in detail elsewhere [38]. In short, emission inventories were compiled for 2000, 2011, and 2018, using data on both local sources and long-range transport, and concentrations for intermediary years interpolated and adjusted for meteorological variations. Local emissions were modelled using dispersion models, while long-range transported emissions were modelled using a bias-corrected chemical transport model. Particle levels were validated through comparison with data from quality-controlled monitoring stations representing urban background concentrations, with generally high *R*^2^ values (PM_2.5_ 0.84, PM_10_ 0.61, NO_x_ 0.87). Yearly average exposure levels were assigned to all study participants (QGIS version 3.22, QGIS Development Team) based on individual address history retrieved from Statistics Sweden, which was automatically geocoded and manually checked and corrected. Exposure concentrations were averaged over ten years before enrolment. Participants with missing exposure data for more than two years were excluded. Concentrations of coarse particles (PM_2.5–10_) were calculated by subtracting PM_2.5_ from PM_10_.

Co-exposures were assessed analogously. Residential exposure to road traffic noise levels were based on traffic intensity data for 2000, 2011, and 2018 and modelled using techniques described previously [2]. Briefly, the 24-h equivalent sound pressure level (L_Aeq, 24 h_) from road traffic was estimated at the most exposed façade, on a dynamic grid with resolutions up to 25 m [55], and averaged over ten years before enrolment. Greenness was calculated as normalised difference vegetation index (NDVI) from satellite data on a 25 × 25 m grid [58]. Pixels obscured by cloud coverage were replaced by a 5-year moving average. Yearly average NDVI values of all non-water surfaces within a 500 m radius were attributed to each participant based on their residential address and averaged over 10 years before enrolment.

### Assessment of renal function and injury

Serum creatinine and cystatin C were measured at enrolment after over-night fasting. Serum creatinine was analysed at each site in fresh blood samples using an enzymatic colorimetric method. Serum cystatin C was analysed using a Gentian Cystatin C reagent in EDTA plasma from frozen samples taken after fasting (Alinity C, Abbott Laboratories, U.S.A.). eGFR was calculated using the CKD-EPI 2021 formulae without race, both combining creatinine and cystatin C and using creatinine and cystatin C separately [32].

Serum uric acid was analysed in fresh fasting plasma samples from participants recruited in Gothenburg, Malmö, and Umeå (*n* = 12 113), using hospital equipment (Roche Cobas 6000/8000). For the remaining serum biomarkers (KIM-1, MCP-1, IL-6, IL-18, MMP-2, MMP-7, MMP-9, and FGF-23), concentrations were obtained in a subsample of participants from all sites (for KIM-1, IL-18, FGF-23, MMP-7, *n* = 4944; for MCP-1, MMP-2, MMP-9, *n* = 5017) using Proximity Extension Assay with O-link Target 96 Cardiovascular II and III panels (Olink Proteomics, Uppsala, Sweden). These biomarker analyses were performed in EDTA plasma and obtained as log_2_-transformed normalised protein expression (NPX) to minimise intra- and inter-assay variation.

### Statistical analysis

We primarily analysed eGFR as a logarithmically transformed continuous outcome, to fully utilise the data while minimising noise at higher eGFR values. Secondarily, we analysed eGFR after dichotomisation at the age and gender specific 10th percentile. We did not choose eGFR 60 mL/min/1.73 m^2^ as cut-off point since very few participants had an eGFR below this level. The biomarkers were analysed as logarithmically transformed continuous outcomes. Linear regression models were fitted with exposure to air pollutants along with four different sets of covariates as independent variables, with logarithmically transformed eGFR or serum concentrations of biomarkers as dependent variables. For low eGFR (eGFR dichotomised at the age and gender specific 10th percentile), robust Poisson models [87] were used instead.

The covariate sets were constructed in a stepwise manner with increasing adjustment using a directed acyclic graph (Figure S1) based on a priori assumptions. The first set included only age, gender, and enrolment site. Age and gender were omitted from the regression model in the analyses of prevalence of low eGFR, as these were included in the definition of the outcome. The second set also included socioeconomic and behavioural risk factors, i.e., highest completed education level (in three categories: compulsory schooling or less, secondary schooling, or tertiary education), civil or cohabitation status (married, in registered partnership, or co-habiting; unmarried and living alone; divorced; or widowed), country of birth (Sweden or any other country), smoking (both as a categorical, i.e., current smoker, former smoker, or never-smoker, and as a continuous variable, lifetime pack-years), physical activity (four categories), alcohol consumption (five categories based on self-reported frequency of alcohol consumption), body-mass index (BMI; categorised according to WHO classes into non-overweight, overweight, or obese), and area-level low-income proportion (defined as proportion of low-income earners in participants’ demographic statistical area [DeSO] area, averaged over ten years before enrolment). Data on proportion of low-income earners, defined as earning less than the national lower income quartile, were obtained per DeSO area from Statistics Sweden for 2000, 2011, and 2018 and linearly interpolated to intermediate years. The third set also included potential mediators, i.e., hypertension (defined as self-reported doctor’s diagnosis or current antihypertensive medication) and diabetes mellitus (based on self-report, categorised as no diabetes, doctor’s diagnosis or current non-insulin treatment, or current insulin treatment). The fourth set also included co-exposures, i.e., long-term road traffic noise (day-evening-night noise level, L_den_) and greenspace (NDVI within 500 m) exposure at the residential address. We considered the second set our main model and excluded participants with incomplete data on these covariates.

Due to high correlations between pollutants (Table 2), we did not conduct multi-pollutant analyses. We explored effect modification by stratification based on gender, age, smoking status, BMI, diabetes, and hypertension. We explored exposure–response relationships by comparing each quarter of exposure with the lowest exposure quarter, and trends across exposure quarters were evaluated with χ^2^ tests for linear trends. As a sensitivity analysis, we performed site-specific analyses, effectively allowing interactions with site for all covariates, which were combined by random effects meta-analyses. In sensitivity analyses probing model specification, we added adjustment for estimated total protein intake (g/day, derived from self-reported dietary data), as high protein intake falsely increases creatinine levels unrelated to renal function, and estimated total alcohol intake (g/day, self-reported), to reduce the risk of residual confounding from alcohol consumption. Further sensitivity analyses investigated exposure assessment, by varying the averaging time window (a dynamic time window covering 2 years before enrolment, accepting no missing exposure data, and a static time window covering 2000‒2015 regardless of enrolment year), as well as the assessment of renal function, by varying the quantile cut-off defining a low eGFR (5%, 25%, and 50%) and analysing associations with creatinine- and cystatin C-based eGFRs separately. As a post hoc sensitivity analysis, we analysed associations with the prevalence of hyperfiltration, defined as eGFR above the age and gender specific 90th percentile.

All analyses were performed in R 4.2 with base and *tidyverse* functions. The ‘geeglm’ function from the *geepack* package was used to fit the robust Poisson regression models, and the ‘dsl’ function from the *metRology* package for the meta-analyses between sites [17, 26, 60].

## Results

Of the 30 154 SCAPIS participants we included 24 729 participants (82%, see Figure S2 for a flow-chart of included and excluded study participants) with exposure and covariate data for the main regression model. Among the included participants, 51% were women, the mean age was 57.5 years, and 51% were never-smokers. Characteristics of the study population per quarter of PM_2.5_ exposure are presented in Table 1. Area-level percentage of low-income earners, proportion unmarried or not cohabiting, prevalence of smoking, and prevalence of high alcohol consumption were higher in the highest exposure quarter. The excluded participants were of similar age and gender composition as the included but came from areas with a slightly higher percentage of low-income earners and were more likely to have low education, to be foreign-born, to smoke, to be obese, or to have diabetes.

**Table 1:** Characteristics of the participants included in the analyses, subdivided per PM_2.5_ exposure quarter

	**All**	**Q1**	**Q2**	**Q3**	**Q4**	**Excluded**^a^
**PM**_**2.5**_, mean (range) [µg/m^3^]	6.2 (3.5–13.4)	5.0 (3.5–5.4)	5.7 (5.4–6.2)	6.7 (6.2–7.4)	8.9 (7.4–13.4)	6.2 (3.5–12.8)
**N**	24 729	6196	6241	6193	6099	5425
**Age**, mean (SD)	57.5 (4.4)	57.5 (4.4)	57.5 (4.37)	57.5 (4.3)	57.7 (4.3)	57.4 (4.3)
**Gender**, % female	51.2	50.5	50.4	50.8	53.2	52.4
**Low-income proportion**, mean area-level %^b^	23.7	20.8	23.0	22.3	28.8	27.3
**Education**, % without secondary schooling	8.8	8.6	8.0	8.4	10.1	12.7
**Civil status**, % neither married nor cohabiting	25.6	18.0	25.0	28.9	30.6	31.1
**Foreign-born**, %	14.1	7.6	12.2	16.7	20.0	28.2
**Smokers**, % current	11.8	7.9	10.0	13.5	15.8	21.2
**Leisure-time physical** **activity**, % sedentary	11.5	10.9	10.1	11.5	13.5	15.5
**Alcohol**, % more than once per week	38.2	32.6	38.4	41.5	40.3	31.9
**Obesity**, % BMI ≥ 30	20.7	21.2	20.0	19.6	22.0	25.2
**Hypertension**, %	22.6	24.0	21.4	21.2	23.8	23.8
**Diabetes**, % (% of whom insulin-treated)	4.2 (29.0)	4.4 (33.7)	3.8 (29.5)	3.9 (27.4)	4.5 (25.0)	5.9 (25.0)
**Residential road traffic** **noise**, mean day-evening- night level [dB] (SD)	55.6 (7.93)	49.5 (8.27)	54.8 (7.18)	58.3 (6.32)	59.6 (5.49)	56.1 (7.87)
**Greenness**, mean NDVI within 500 m (SD)	0.47 (0.12)	0.55 (0.09)	0.5 (0.1)	0.45 (0.11)	0.39 (0.11)	0.46 (0.12)
**eGFR**, median (IQR)	99.2 (18.7)	98.4 (19.0)	98.9 (18.8)	100.9 (17.8)	98.5 (19.2)	99.3 (19.5)

^a^Includes individuals excluded due to missing exposure, outcome, or any of the covariates for the main model

^b^Low-income earners were defined as individuals with annual income below than the lower national income quartile

Median PM_2.5_ exposure was 6.2 µg/m^3^ (5th–95th percentile 4.5–9.8). Median exposure, interquartile ranges, and correlations with total and local PM_2.5_ for each assessed pollutant are presented in Table 2. Median eGFR was 99.2 mL/min/1.73 m^2^ (5th–95th percentile 73.4–119.8 mL/min/1.73 m^2^) and prevalence of eGFR < 60 mL/min/1.73 m^2^ was 1.0% (*n* = 245).

**Table 2:** Median long-term exposure (10 years before enrolment), interquartile ranges (IQR), and Pearson correlations with PM2.5 (total) and PM2.5 (local) for each air pollutant

			**Pearson correlations**	
	**Median [mg/m**^**3**^**]**	**IQR [mg/m**^**3**^**]**	**PM**_**2.5**_** (total)**	**PM**_**2.5**_** (local)**
**PM**_**2.5**_** (total)**	6.2	2.03	–	0.75
**PM**_**2.5**_** (local)**	0.8	0.79	0.75	–
**PM**_**10**_** (total)**	11.5	5.74	0.85	0.79
**PM**_**10**_** (local)**	2.1	1.58	0.35	0.78
**PM**_**2.5–10**_** (total)**	5.3	2.92	0.55	0.65
**PM**_**2.5–10**_** (local)**	1.1	1.18	0.08	0.53
**NO**_**x**_** (total)**	12.5	13.20	0.79	0.92
**NO**_**x**_** (local)**	8.7	10.67	0.69	0.92

**Fig. 1:**
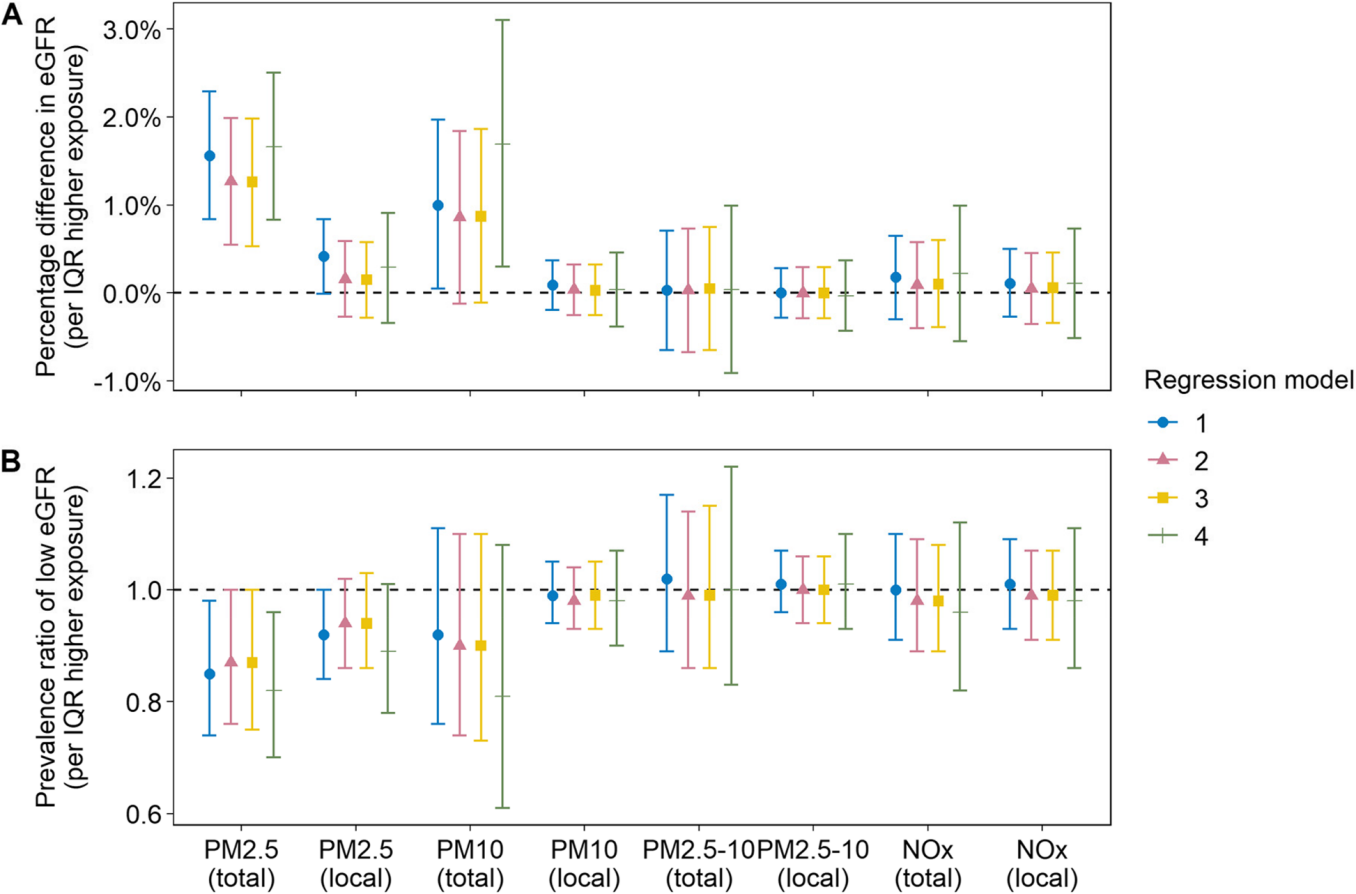
Associations between exposure to different air pollutants and **A)** eGFR, and **B)** prevalence of a low eGFR (below age- and gender-specific tenth percentile) per interquartile range (IQR) higher exposure. Model 1 included only age, gender, and study site (age and gender were omitted in analyses of prevalence of a low eGFR). Model 2 included all covariates from model 1 as well as sociodemographic and behavioural risk factors (area-level income, education level, civil or cohabitation status, immigration status, physical activity, alcohol consumption, and BMI). Model 3 included all covariates from model 2 as well as hypertension and diabetes mellitus diagnoses. Model 4 included all covariates from model 3 as well as road traffic noise and greenness exposure. (Model 1‒2, *n* = 24 729; model 3, *n* = 24 464; model 4, *n* = 24 400.)

Associations between air pollution exposure and eGFR and prevalence of a low eGFR are presented in Fig. 1 and Table S1. After adjustment for site, age, gender, and socioeconomic and behavioural risk factors (model 2), an interquartile range (IQR, 2.03 µg/m^3^) higher long-term exposure to PM_2.5_ was associated with a 1.27% (95% CI 0.55, 1.99) higher eGFR, or a 13% lower prevalence of a low eGFR (prevalence ratio [PR] 0.87, 95% CI 0.76, 1.00). These estimates were robust to covariate adjustment, including for potential mediators (hypertension and diabetes, model 3) and co-exposures (noise and greenness, model 4), as well as in sensitivity analyses with additional adjustment for estimated protein intake and alcohol consumption (g/day) (Table S2). Exposure to PM_10_ was similarly associated with a higher eGFR, but with a slightly lower point estimate per interquartile range increment of exposure and wider confidence intervals. Locally emitted PM_2.5_ tended to be associated with a higher eGFR and a lower prevalence of low eGFR, while associations with NO_x_, PM_2.5–10_, and locally emitted PM_10_ were null. When analysing the association with prevalence of hyperfiltration, we found elevated PRs for PM_2.5_ (PR 1.14, 95% CI 0.99, 1.31 per 2.03 µg/m^3^), but no associations with the other pollutants.

When higher eGFR cut-offs were chosen the prevalence ratios were slightly closer to the null but with lower statistical uncertainty (e.g., a cut-off at the 25th percentile resulted in PR 0.89, 95% CI 0.82, 0.96, per 2.03 µg/m^3^ [IQR] higher PM_2.5_, Table S3). In separate analyses at each site, associations between PM_2.5_ and eGFR were positive or weakly positive in four out of six sites (Table S4). Combining the site-specific estimates using random-effects meta-analysis resulted in an overall association of 1.38% (95% CI -0.27, 3.04) higher eGFR per 2.03 µg/m^3^ (IQR) higher PM_2.5_. Averaging exposure over two years before enrolment instead of ten years diminished associations slightly, and associations were null when averaging exposure over a static time frame between 2000 and 2015 for all participants regardless of enrolment year (Table S5). Sensitivity analyses with creatinine- and cystatin C-based eGFR separately showed positive associations between PM_2.5_ and both creatinine-based eGFR and with cystatin C-based eGFR (1.44%, 95% CI 0.75, 2.12, higher eGFR_creatinine_ per 2.03 µg/m^3^ [IQR] higher PM_2.5_; 0.96%, 95% CI 0.12, 1.82, higher eGFR_cystatin C_, per 2.03 µg/m^3^ [IQR] higher PM_2.5_; Table S6).

**Fig. 2:**
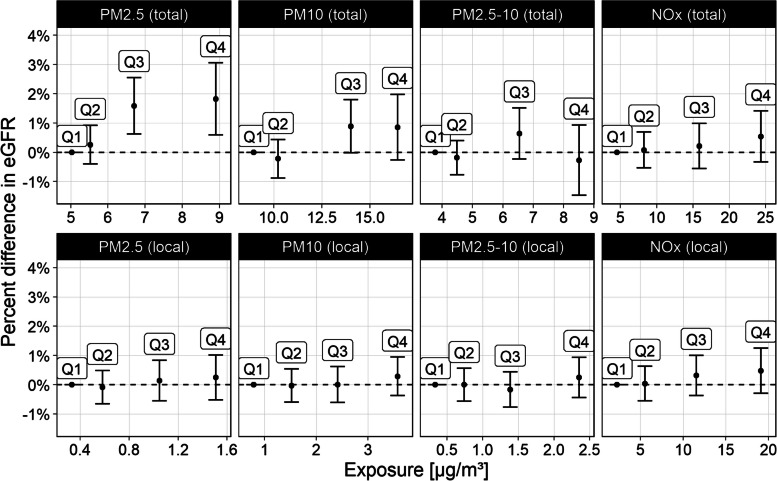
Percentage difference in renal function for exposure quarters 2 to 4 (Q2–4) compared to the first quarter (Q1). Bars are placed at the median exposure of each quarter

An exposure–response pattern was visible for PM_2.5_ (total), with stronger associations in the highest quarter compared to the lowest (Q4 vs. Q1 1.82%, 95% CI 0.60, 3.06, higher eGFR; Fig. 2, Table S7) and a trend across estimates for each exposure quarter (*p* = 0.001). In stratified analyses, we found no association between PM_2.5_ exposure and eGFR among current smokers, but a tendency towards stronger associations among participants with a high BMI or diabetes (Figure S3). However, we found no evidence of an interaction effect for any of the assessed potential effect modifiers.

**Fig. 3:**
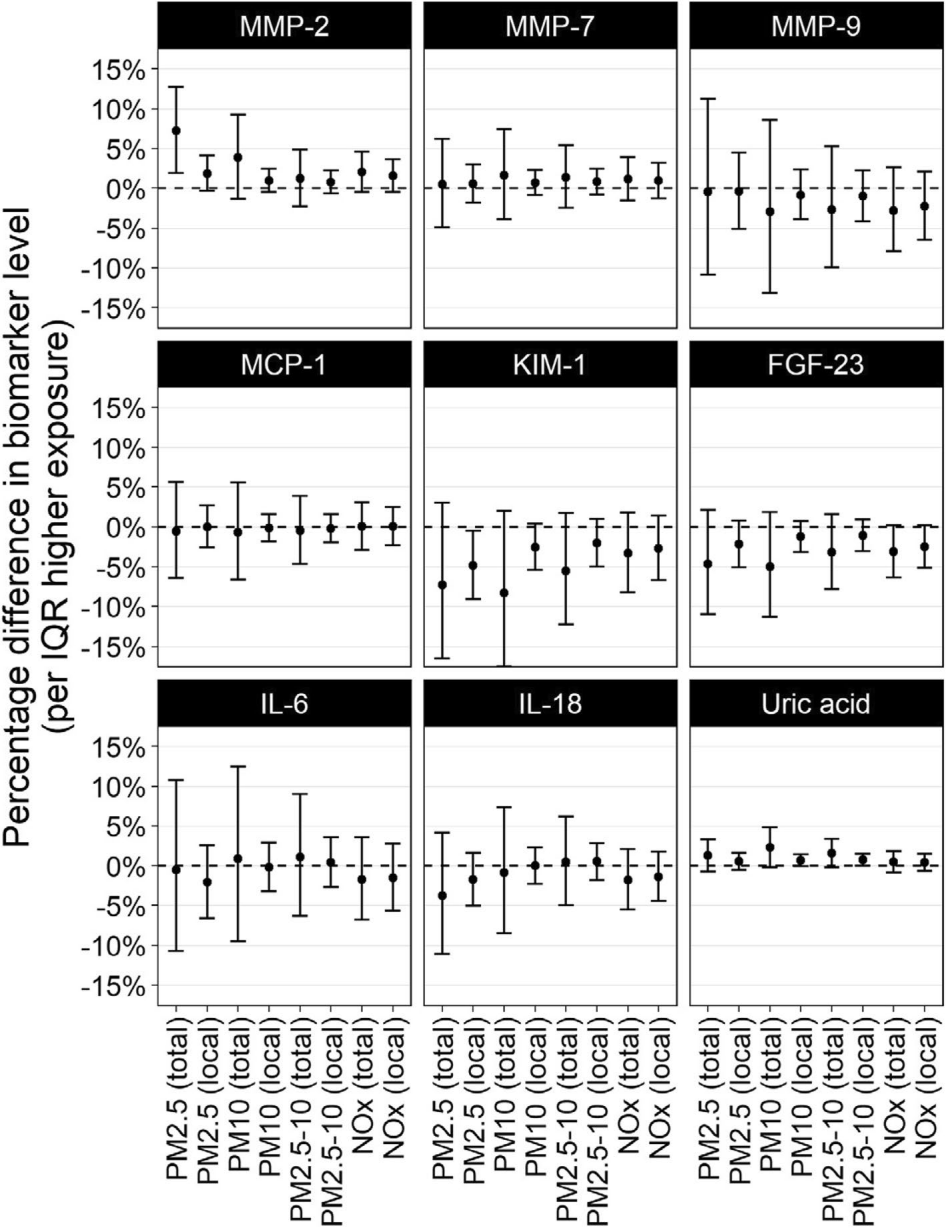
Associations between long-term exposure to air pollutants and concentrations of serum biomarkers of renal injury, adjusted for age, gender, site, and socioeconomic and behavioural risk factors (model 2)

We observed an association between PM_2.5_ and MMP-2, where an IQR (2.03 µg/m^3^) higher exposure to PM_2.5_ (total) was associated with 7.21% (95% CI 1.94, 12.75) higher MMP-2 levels (Fig. 3, Table S8). For MMP-2, we also observed an exposure–response pattern (16.02%, 95% CI 3.47, 30.10, higher MMP-2 in Q4 vs. Q1, *p*-value for linear trend across exposure quarters 0.028, Table S9). In site-specific analyses, the association between PM_2.5_ and MMP-2 was positive in five of the six sites, with a pooled effect estimate of 6.84% (95% CI 1.70, 11.97) per 2.03 µg/m^3^ [IQR] PM_2.5_ (total) (Table S10).

The association between PM_2.5_ and PM_10_ and serum uric acid tended towards the positive (1.28%, 95% CI -0.70, 3.30, per 5.74 µg/m^3^ [IQR] higher PM_10_ (total), Table S8), but there was no indication of an exposure–response pattern (Table S9). However, associations were consistent across the three sites at which serum uric acid data were available, with a pooled estimate 2.38% (95% CI ‒0.09, 4.84) per 5.74 µg/m^3^ [IQR] higher PM_10_ (total), with comparably elevated estimates for all assessed pollutants (Table S10). On the contrary, FGF-23 and KIM-1 were slightly negatively associated with air pollution exposure. Comparing the highest exposure quarter to the lowest, KIM-1 and IL-6 were negatively associated with PM_2.5_ (local) (Q4 vs. Q1 ‒8.32%, 95% CI ‒15.34, ‒0.71, *p*-value for linear trend 0.038, and ‒10.14%, 95% CI ‒17.29, ‒2.36, *p*-value for linear trend 0.018, respectively, Table S9). There were no clear associations between any of the assessed pollutants and serum concentrations of IL-18, MCP-1, MMP-7, or MMP-9.

## Discussion

In this large sample of middle-aged men and women from the general population of six Swedish cities, long-term exposure to PM_2.5_ was associated with a slightly higher renal filtration rate and a lower prevalence of a low eGFR (defined as an eGFR below the age and gender specific 10th percentile). This association exhibited an exposure–response pattern and was robust to adjustment for risk factors and co-exposures, including potential confounders and mediators. Other assessed pollutants were not consistently associated with renal function. Furthermore, exposure to PM_2.5_ was associated with increased serum concentrations of MMP-2 and possibly uric acid, while associations with KIM-1 and FGF-23 instead tended to be negative and associations with IL-6 were consistently null.

To the best of our knowledge, our study is the first to investigate associations between long-term exposure to air pollution and renal function in a large general population sample outside of East Asia or the Americas. One of the first studies investigating air pollution and renal function was a cross-sectional study in a sample of Boston stroke patients, which found a 3.9 mL/min/1.73 m^2^ (95% CI 1.0, 6.7) lower eGFR in patients residing within 50 m of a major roadway compared to patients residing > 1000 m from a major roadway, although this study was not able to differentiate between air pollution and other effects of traffic [43]. A larger, longitudinal study in US veterans found an average additional yearly decrease in eGFR of 0.6 mL/min/1.73 m^2^ (95% CI 0.40, 0.79) per 2.1 µg/m^3^ higher exposure to fine particles (PM_2.5_), in a population with mean PM_2.5_ exposure of 11.4 µg/m^3^ [44]. Similar results have also been reported from some more recent studies [16, 40, 41, 70, 79, 85].

A few studies, however, have found opposite or null associations between air pollution and eGFR, some of which in the same direction as our findings. In a cross-sectional analysis of the Jackson Heart Study (JHS), Weaver et al. [81] found a tendency towards a positive association between PM_2.5_ exposure and eGFR (0.8 mL/min/1.73 m^2^, 95% CI -0.4, 1.9, higher eGFR per 1 µg/m^3^). A recent cross-sectional study in a large Taiwanese population found that exposure to pollutants commonly found in fresh exhaust (i.e., CO, NO, and NO_x_) were associated with a higher eGFR, although PM_2.5_, ozone, and sulphur dioxide were associated with a lower eGFR [73]. A smaller, Flemish study, at only slightly higher exposure levels than in the SCAPIS population, found no relation between PM_2.5_ and eGFR either cross-sectionally or longitudinally (Feng et al. 2021).

The inconsistency compared to most previous studies may be due to the high baseline eGFR in the study population and the low exposure level. Physiologically, it is possible that exposure to low levels of air pollution may cause increased eGFR, especially at the high eGFR levels present in this renally healthy population. We did observe a small increment in the prevalence rate of hyperfiltration, associated with a higher PM_2.5_ exposure. For comparison, glomerular hyperfiltration is established in early type 1 and type 2 diabetes mellitus and is hypothesised to predisposed to more rapid progression of kidney damage [76]. Glomerular hyperfiltration has been associated with higher risks of cardiovascular mortality and morbidity [63]. Air pollution exposure can be compared to cigarette smoking, which is believed to increase creatinine clearance and cause proteinuria [27, 45]. The null associations between air pollution and eGFR among current smokers in this study is consistent with this hypothesis. Notably, however, in our study the association between exposure and a higher eGFR was also found for cystatin C-based eGFR (Table S6).

It cannot be excluded that our findings are due to residual or unmeasured confounding, despite rigorous confounding adjustment and little difference across regression models or sensitivity analyses. The E-value for the dichotomous outcome (eGFR below the 10th age and gender specific percentile) was 1.5, meaning that a relatively common but unmeasured or residual confounder need to be associated with both the exposure and the outcome by at least a 50% higher prevalence, above and beyond the potential confounders already adjusted for, in order to fully explain our findings [15]. Although relatively small, this figure can be compared to frequent alcohol consumption (≥ 4 times per week), which was associated with a 70% higher prevalence of low eGFR when included as a covariate in our analyses and with a 40% higher prevalence of above-median air pollution exposure after adjustment for all other covariates in the main model.

We noted a consistent association between PM_2.5_ exposure and serum concentration of MMP-2 (7.21%, 95% CI 1.94, 12.75, higher MMP-2 concentrations in serum per 2.03 µg/m^3^ [IQR] higher PM_2.5_ exposure, Table S8). This finding is important, as it may provide a link between PM_2.5_ exposure and its impacts on cardiorenal outcomes. However, since it was the only consistent association observed among the biomarkers, the risk of a chance finding cannot be excluded. While, to our knowledge, the present study is the first to investigate an association between PM_2.5_ exposure and MMP-2 in a general population sample, in vitro studies have reported a cellular upregulation of MMP-2 in the response to oxidative stress caused by air pollutants [34, 47]. Notably, though, these studies showed that both MMP-2 and MMP-9 mediated oxidative stress effects of particle exposure, while in this study we found no association between exposure to any air pollutant and serum concentrations of MMP-9.

A potential route for PM_2.5_-related induction of MMPs is oxidative stress, which increases MMP levels through increases in both expression and activation of pro-MMPs [48]. MMP-2 and MMP-9 play various roles in the renal disease progression, contributing to loss of glomerular cell junctions, epithelial-to-mesenchymal transition, and increased renal fibrosis, accelerating the decline in renal function [59]. A prospective cohort study in non-diabetic patients with coronary artery disease concluded that elevated serum MMP-2, -3, and -9 were predictive of more rapid eGFR decline [29]. MMPs have also been widely considered to play an important role in the atherosclerotic disease process, not least as biomarkers of plaque instability and rupture [48, 51, 56]. The relationship between air pollution and serum MMP-2 is therefore especially interesting in light of our recent finding, in the same study population, of an association between PM_2.5_ exposure and non-calcified coronary artery plaques, which is a radiological characteristic of early or vulnerable atherosclerosis [37].

We also noted a tentative association between air pollution exposure, in particular PM_10_ and PM_2.5‒10_, and serum uric acid. Increased serum uric acid, even below the threshold for clinical hyperuricaemia, is an independent risk factor for accelerated eGFR loss and CKD incidence in the general population [5, 39, 82], as well as for general and cardiovascular mortality [18, 52]. The primary route of uric acid elimination is proximal tubular secretion, and the risk increases for CKD associated with elevated serum uric acid have been shown to be independent of baseline GFR [53, 64]. Being produced by xanthine oxidase, uric acid has also been discussed as a marker or product of oxidative stress, with one study showing a correlation between serum uric acid and LDL oxidation [13]. The tentative association between air pollution exposure and uric acid in our study is in line with the small number of previous studies at much higher exposure levels. A prospective cohort study in traffic police in a major Chinese city showed that a 10 µg/m^3^ higher long-term PM_10_ exposure was associated with 11.54% (95% 8.14, 14.93) higher serum uric acid [74]. Another Chinese population-based study recently reported similar associations for PM_2.5_ [31]. Similar results were reported from a longitudinal study of short-term PM_2.5_ exposure [23]. However, further studies are needed to draw conclusions on the relationship between air pollution and uric acid and its potential clinical implications.

The negative association noted with KIM-1 is surprising. KIM-1 is a membrane protein expressed in the apical membrane of proximal tubular cells in response to acute kidney injury, where it promotes cell phagocytosis, tubular injury repair, and inhibition of renal tubular inflammation. However, in the context of CKD, KIM-1 has instead been shown to promote inflammation, fibrosis, and apoptosis, in part by promoting MCP-1 excretion which contribute to macrophage recruitment [71]. Plasma concentrations of KIM-1 have been shown to closely reflect urinary levels, correlate with albumin excretion, and identify patients with CKD of various aetiologies [65]. Recently, a cohort study in healthy participants from the general Swedish population showed that a standard deviation higher plasma KIM-1 was associated with future risk of hospitalisation for impaired renal function (hazard ratio 1.43, 95% CI 1.18–1.74) [68]. Similar results have been reported from several studies in type II diabetes mellitus patients [9, 14, 35].

The primary strengths of this study include our large study population at low exposure levels, the extensive covariate and biomarker data available for SCAPIS participants, and the comparatively high validity of the modelled annual average concentrations of PM_2.5_, PM_10_, and NO_x_ [38]. On the other hand, the conclusions drawn from this study are limited by the narrow exposure contrasts, the strong relation between exposure and enrolment site, and the comparatively healthy SCAPIS population with generally good renal function. Most of the within-site variability was accounted for by enrolment year, owing to the decreasing trend in PM_2.5_ concentrations in Sweden, with enrolment site and year together explaining 94% of the exposure variability. There is, of course, still the possibility for measurement error affecting our results; however, if this is non-differential it would bias associations towards the null and is therefore unlikely to explain the inverse associations we observe. Previous studies have shown that air pollution concentrations at the residential address is a valid proxy for total personal exposure [33]. Recently, it was shown that the exposure assessment at the residential address may be biased for certain groups, particularly among individuals who commute farther than 10 miles [42], which is the case for few of the SCAPIS participants. The narrow exposure contrasts are thus most likely reflective of relatively homogenous exposure levels within the source population.

Another strength of our study is the use of both creatinine and cystatin C to calculate a combined eGFR, which is superior to GFR estimates based on only one of the two biomarkers [25, 72]. This is important, as eGFR is a less reliable estimator of renal function in healthy individuals [75]. Creatinine levels can be falsely inflated for individuals with high protein intake, however adjustment for protein intake (Table S2) did not affect our results. A limitation was that we were unable to investigate the association between air pollution and proteinuria, albuminuria, or albumin-creatinine ratio, which are also integral markers of renal disease. A few studies have investigated a potential association between air pollution exposure and proteinuria, with most, but not all, finding such an association [8, 19, 41, 57, 81]. To better characterise the kidney disease progression and more closely resemble the clinical CKD diagnosis, future studies should, if possible, also include measures of urinary albumin or protein excretion. Lastly, an important limitation of the current study is its cross-sectional design. Follow-up examinations of SCAPIS participants are planned to be completed in 2026, which will enable longitudinal analyses in this cohort.

## Conclusions

In this cross-sectional analysis of a large sample of middle-aged men and women in Sweden we found an association between PM_2.5_ exposure and a higher estimated glomerular filtration rate, indicating a potential effect towards increased filtration at low-level exposure in a renally healthy population, which may be an early sign of renal disease. PM_2.5_ exposure was, however, associated with higher serum concentrations of metalloproteinase 2 (MMP-2), an early indicator of renal and cardiovascular pathology. This study is, to the best of our knowledge, the first large-scale population-based investigation of the relationship between air pollution and renal function in Europe and extends our knowledge of this association at the lower end of the exposure spectrum.

### Supplementary Information

Supplementary Material 1.

Supplementary Material 2.

## Data Availability

Due to the sensitive nature of the data, we are not able to share the data used for this study publicly. However, all data are available to researchers upon reasonable request (including an ethical permission) from the SCAPIS consortium (www.scapis.org).
