# Supplementary material for: Long-term ambient air pollution exposure and renal function and biomarkers of renal disease
Source: Environ Health. 2024 Aug 9;23:67. doi: 10.1186/s12940-024-01108-9 (PMC11313149; doi:10.1186/s12940-024-01108-9)

**Figure S1.** Directed acyclic graph (DAG).


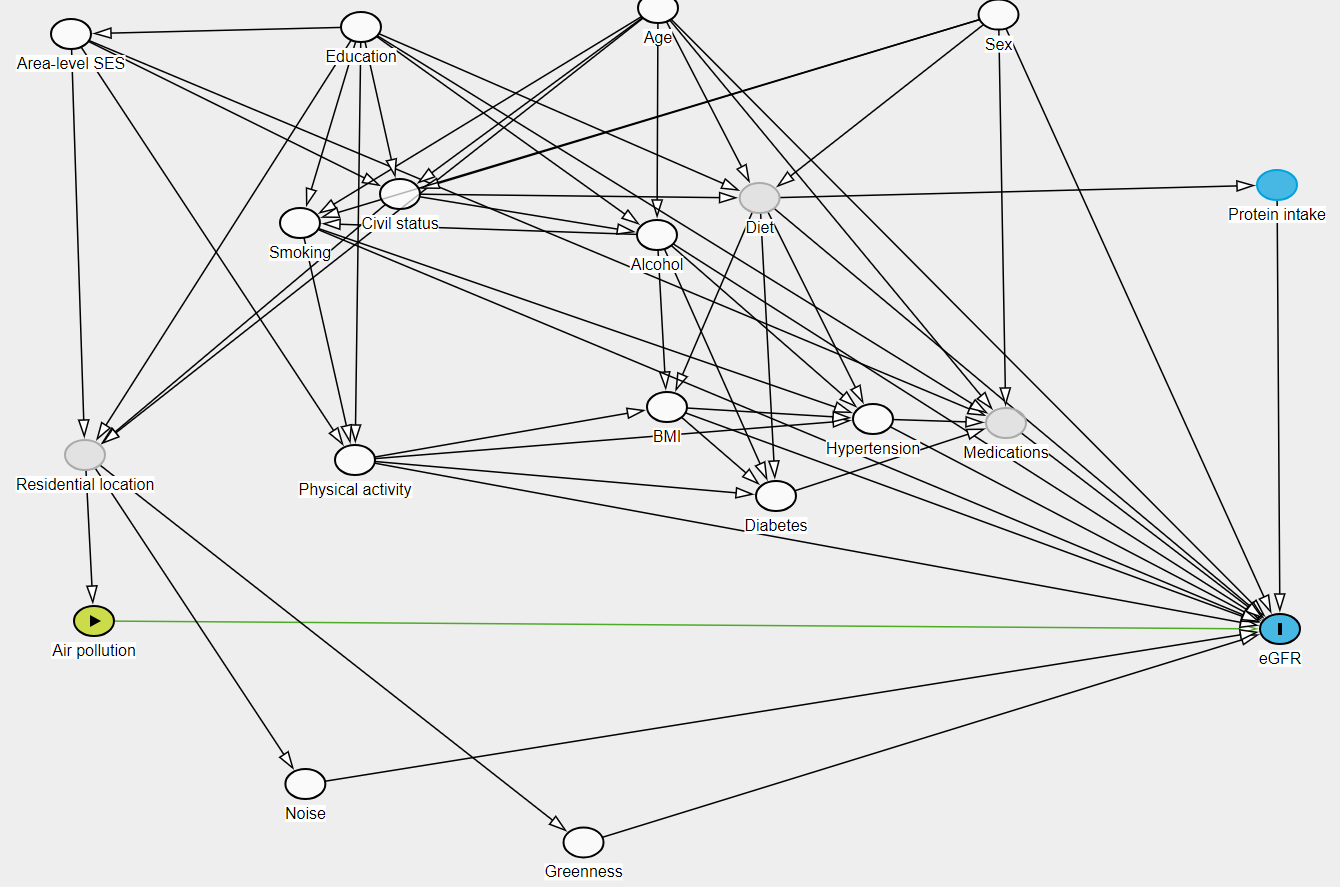


**Figure S2.** Flow-chart of included and excluded participants.


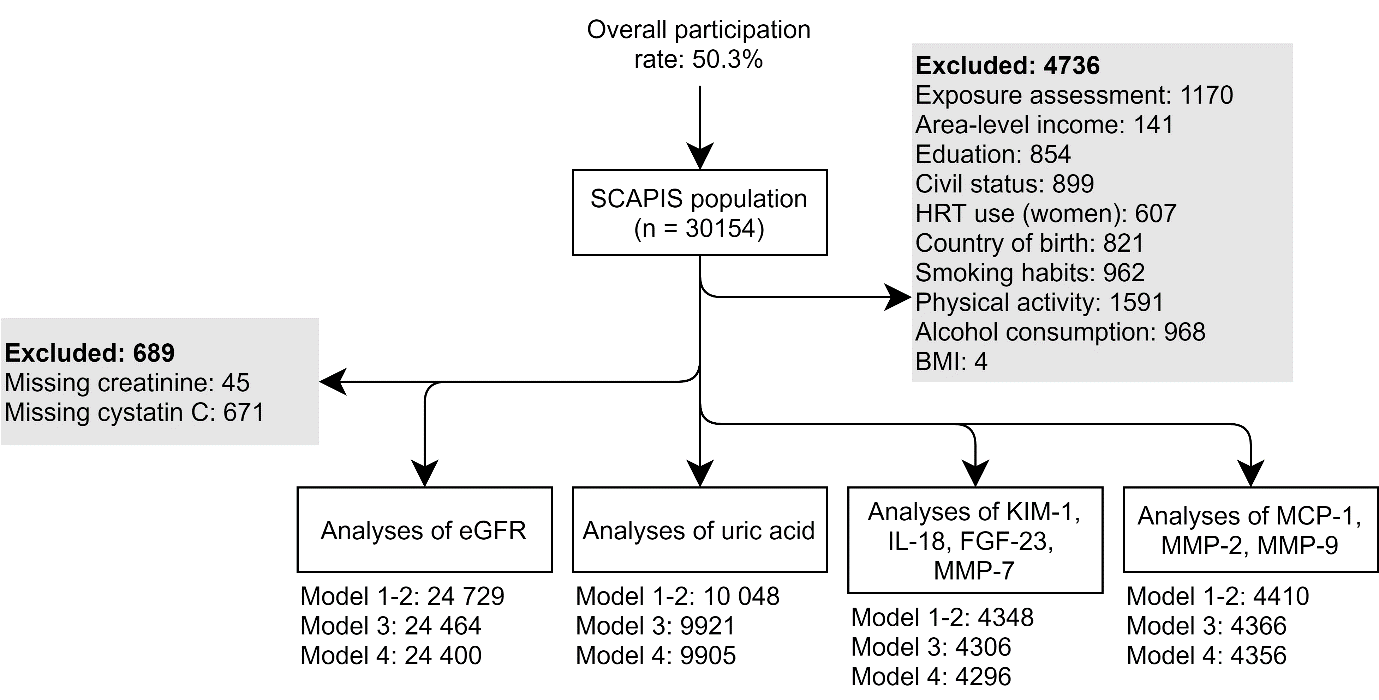


**Figure S3.** Tree-plot of assessed effect modifiers, with number of participants (N), percentage difference in eGFR per interquartile range (IQR) higher PM_2.5_ (total) and p-value for the interaction term.


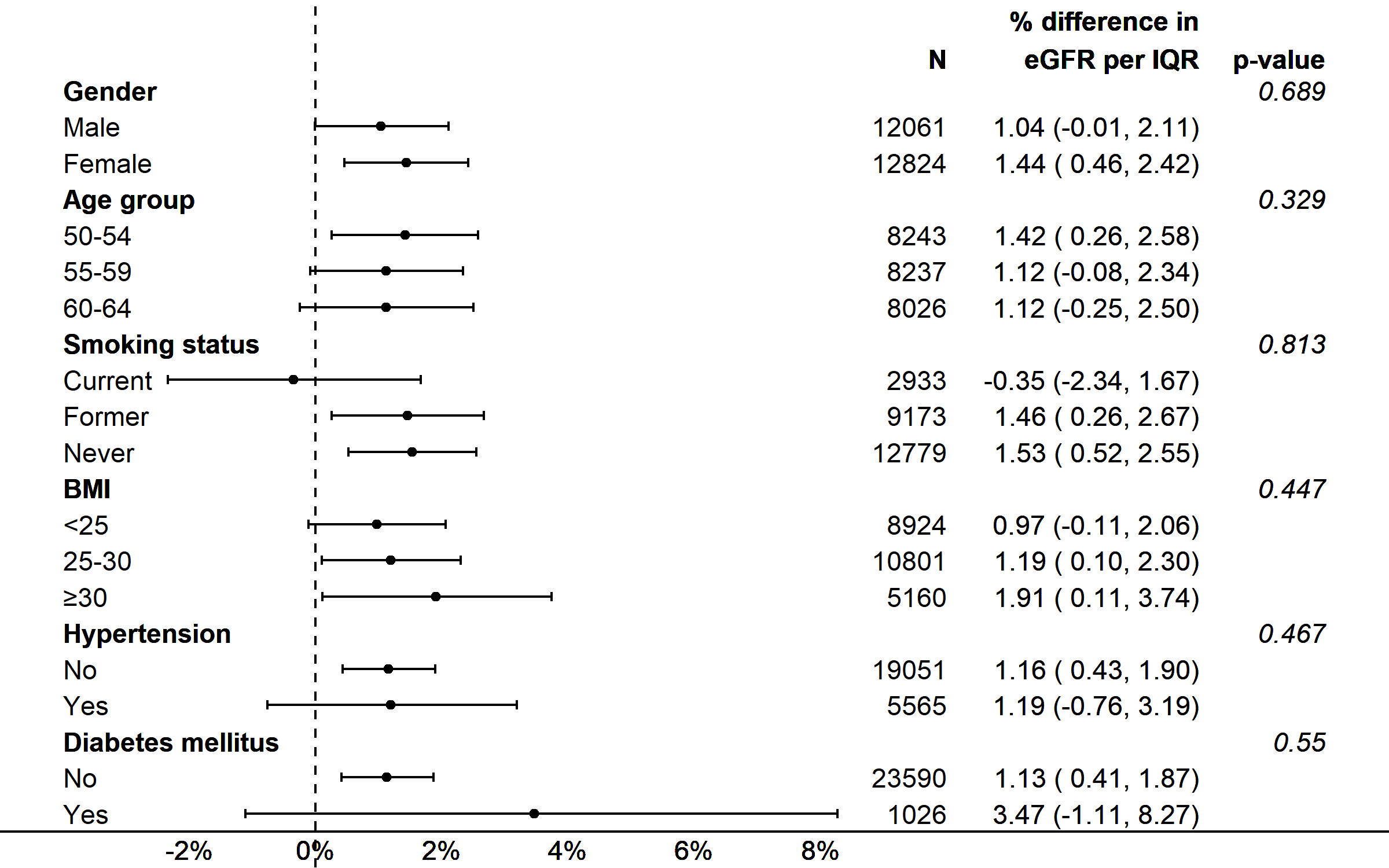

Supplement: Supplementary file 1 — Supplementary Material 1. [file 12940_2024_1108_MOESM1_ESM.docx]
