# Supplementary material for: Long-term ambient air pollution exposure and renal function and biomarkers of renal disease
Source: Environ Health. 2024 Aug 9;23:67. doi: 10.1186/s12940-024-01108-9 (PMC11313149; doi:10.1186/s12940-024-01108-9)
Supplement: Supplementary file 2 — Supplementary Material 2. [file 12940_2024_1108_MOESM2_ESM.docx]

**Table S1**. Associations between air pollution exposure and eGFR, and air pollutants and prevalence of low eGFR (below age and gender specific 10^th^ percentile).

| **Model** | **PM_2.5_ (total)** | **PM_2.5_ (local)** | **PM_10_ (total)** | **PM_10_ (local)** | **PM_2.5–10_ (total)** | **PM_2.5–10_ (local)** | **NO_x_ (total)** | **NO_x_ (local)** |
| --- | --- | --- | --- | --- | --- | --- | --- | --- |
| Difference in eGFR per interquartile range (IQR) higher exposure, % difference (95% confidence intervals) | | | | | | | | |
| 1 | 1.56 (0.84, 2.29) | 0.42 (-0.01, 0.84) | 1.00 (0.05, 1.97) | 0.09 (-0.19, 0.37) | 0.03 (-0.65, 0.71) | 0.00 (-0.28, 0.28) | 0.18 (-0.30, 0.65) | 0.11 (-0.27, 0.50) |
| 2 | 1.27 (0.55, 1.99) | 0.16 (-0.27, 0.59) | 0.86 (-0.12, 1.84) | 0.04 (-0.25, 0.32) | 0.03 (-0.67, 0.73) | 0.00 (-0.29, 0.29) | 0.09 (-0.40, 0.58) | 0.05 (-0.35, 0.45) |
| 3 | 1.26 (0.53, 1.98) | 0.15 (-0.28, 0.58) | 0.87 (-0.11, 1.86) | 0.03 (-0.25, 0.32) | 0.05 (-0.65, 0.75) | 0.00 (-0.29, 0.29) | 0.10 (-0.39, 0.60) | 0.06 (-0.34, 0.46) |
| 4 | 1.66 (0.83, 2.50) | 0.29 (-0.34, 0.91) | 1.69 (0.30, 3.10) | 0.04 (-0.38, 0.46) | 0.04 (-0.91, 0.99) | -0.03 (-0.43, 0.37) | 0.22 (-0.55, 0.99) | 0.11 (-0.51, 0.73) |
| Difference in prevalence of a low eGFR per interquartile range (IQR) higher exposure, prevalence ratios (95% confidence intervals) | | | | | | | | |
| 1 | 0.85 (0.74, 0.98) | 0.92 (0.84, 1.00) | 0.92 (0.76, 1.11) | 0.99 (0.94, 1.05) | 1.02 (0.89, 1.17) | 1.01 (0.96, 1.07) | 1.00 (0.91, 1.10) | 1.01 (0.93, 1.09) |
| 2 | 0.87 (0.76, 1.00) | 0.94 (0.86, 1.02) | 0.90 (0.74, 1.10) | 0.98 (0.93, 1.04) | 0.99 (0.86, 1.14) | 1.00 (0.94, 1.06) | 0.98 (0.89, 1.09) | 0.99 (0.91, 1.07) |
| 3 | 0.87 (0.75, 1.00) | 0.94 (0.86, 1.03) | 0.90 (0.73, 1.10) | 0.99 (0.93, 1.05) | 0.99 (0.86, 1.15) | 1.00 (0.94, 1.06) | 0.98 (0.89, 1.08) | 0.99 (0.91, 1.07) |
| 4 | 0.82 (0.70, 0.96) | 0.89 (0.78, 1.01) | 0.81 (0.61, 1.08) | 0.98 (0.90, 1.07) | 1.00 (0.83, 1.22) | 1.01 (0.93, 1.10) | 0.96 (0.82, 1.12) | 0.98 (0.86, 1.11) |

**Table S2.** Associations between air pollutants and percentage difference (95% confidence intervals) in eGFR with adjustment for the main covariate model with addition of estimated protein intake (g/day) and estimated alcohol consumption (g/day).

|  | **Percent difference in eGFR** | |
| --- | --- | --- |
|  | Protein intake (g/day) | Alcohol consumption (g/day) |
| PM_2.5_ (total) | 1.30 (0.57, 2.04) | 1.27 (0.54, 2.01) |
| PM_2.5_ (local) | 0.18 (-0.25, 0.62) | 0.13 (-0.30, 0.57) |
| PM_10_ (total) | 0.91 (-0.08, 1.91) | 0.79 (-0.20, 1.79) |
| PM_10_ (local) | 0.06 (-0.23, 0.34) | 0.02 (-0.27, 0.30) |
| PM_2.5–10_ (total) | 0.07 (-0.64, 0.78) | -0.03 (-0.73, 0.68) |
| PM_2.5–10_ (local) | 0.02 (-0.27, 0.32) | -0.02 (-0.31, 0.27) |
| NO_x_ (total) | 0.08 (-0.42, 0.58) | 0.02 (-0.47, 0.52) |
| NO_x_ (local) | 0.04 (-0.36, 0.44) | 0.00 (-0.40, 0.40) |

**Table S3.** Associations between air pollutants and prevalence of a low eGFR (95% confidence intervals) using different percentile cut-offs (5^th^ percentile, 25^th^ percentile, and median), with adjustment for covariates in model 2.

| **Percentile** | | **PM_2.5_ (total)** | **PM_2.5_ (local)** | **PM_10_ (total)** | **PM_10_ (local)** | **PM_2.5–10_ (total)** | **PM_2.5–10_ (local)** | **NO_x_ (total)** | **NO_x_ (local)** |
| --- | --- | --- | --- | --- | --- | --- | --- | --- | --- |
| 5^th^ | 0.80 (0.65, 0.97) | | 0.94 (0.83, 1.06) | 0.89 (0.67, 1.19) | 1.00 (0.92, 1.09) | 1.04 (0.85, 1.28) | 1.02 (0.94, 1.11) | 1.02 (0.88, 1.18) | 1.02 (0.91, 1.15) |
| 25^th^ | 0.89 (0.82, 0.96) | | 0.99 (0.94, 1.04) | 0.93 (0.83, 1.04) | 1.00 (0.97, 1.03) | 1.01 (0.93, 1.09) | 1.00 (0.97, 1.04) | 1.00 (0.94, 1.06) | 1.00 (0.96, 1.05) |
| Median | 0.92 (0.88, 0.97) | | 0.98 (0.95, 1.01) | 0.94 (0.88, 1.00) | 0.99 (0.97, 1.01) | 0.99 (0.95, 1.04) | 1.00 (0.98, 1.02) | 0.98 (0.94, 1.01) | 0.98 (0.96, 1.01) |

**Table S4.** Associations between air pollutants and percentage difference in eGFR (95% confidence intervals) per interquartile range (IQR, for the entire study population) higher exposure, for each site and combined using random-effects meta-analysis, with adjustment for covariates in model 2.

| **Site** | **PM_2.5_ (total)** | **PM_2.5_ (local)** | **PM_10_ (total)** | **PM_10_ (local)** | **PM_2.5–10_ (total)** | **PM_2.5–10_ (local)** | **NO_x_ (total)** | **NO_x_ (local)** |
| --- | --- | --- | --- | --- | --- | --- | --- | --- |
| Umeå | -0.19 (-4.05, 3.83) | -0.09 (-1.90, 1.75) | 0.59 (-2.74, 4.02) | 0.22 (-0.76, 1.20) | 0.59 (-1.71, 2.94) | 0.30 (-0.66, 1.27) | 0.42 (-2.68, 3.61) | 0.40 (-2.13, 2.99) |
| Uppsala | -2.09 (-4.82, 0.72) | -0.97 (-2.58, 0.66) | -1.15 (-4.77, 2.60) | -0.34 (-1.45, 0.78) | 0.16 (-2.72, 3.13) | -0.17 (-1.39, 1.06) | -0.48 (-2.10, 1.17) | -0.37 (-1.68, 0.96) |
| Stockholm | 4.13 (2.29, 6.01) | 1.00 (0.01, 1.99) | 3.96 (1.48, 6.51) | 0.72 (0.02, 1.44) | 1.50 (-0.38, 3.42) | 0.75 (-0.03, 1.53) | 0.98 (0.06, 1.91) | 0.76 (0.02, 1.51) |
| Linköping | 4.25 (1.18, 7.40) | 0.48 (-1.28, 2.27) | 0.77 (-1.84, 3.46) | -0.06 (-0.78, 0.66) | -0.28 (-1.88, 1.36) | -0.11 (-0.77, 0.54) | 0.62 (-1.66, 2.94) | 0.43 (-1.40, 2.30) |
| Gothenburg | 0.43 (-0.68, 1.55) | -0.19 (-0.81, 0.42) | -0.06 (-1.47, 1.38) | -0.19 (-0.60, 0.22) | -0.29 (-1.28, 0.70) | -0.20 (-0.62, 0.21) | -0.57 (-1.31, 0.19) | -0.47 (-1.07, 0.14) |
| Malmö | 1.32 (-0.02, 2.68) | 0.66 (-0.40, 1.74) | 1.95 (-1.21, 5.21) | 0.19 (-0.90, 1.30) | -1.30 (-4.37, 1.88) | -0.36 (-1.78, 1.09) | 0.28 (-0.94, 1.50) | 0.15 (-0.83, 1.14) |
| *Total* | *1.38 (-0.27, 3.04)* | *0.20 (-0.35, 0.75)* | *1.04 (-0.40, 2.48)* | *0.04 (-0.26, 0.35)* | *0.01 (-0.68, 0.71)* | *0.00 (-0.31, 0.30)* | *0.13 (-0.52, 0.78)* | *0.08 (-0.43, 0.59)* |

**Table S5.** Associations between air pollutant exposure and percentage difference in eGFR (95% confidence intervals) over different exposure averaging times, i.e., two or five years before enrolment, instead of ten years, or over a static time frame between 2000 and 2015 for all participants regardless of enrolment year.

| **Model** | **PM_2.5_ (total)** | **PM_2.5_ (local)** | **PM_10_ (total)** | **PM_10_ (local)** | **PM_2.5–10_ (total)** | **PM_2.5–10_ (local)** | **NO_x_ (total)** | **NO_x_ (local)** |
| --- | --- | --- | --- | --- | --- | --- | --- | --- |
| Exposure averaged over 2 years before enrolment | | | | | | | | |
| 1 | 1.34 (0.75, 1.94) | 0.31 (-0.06, 0.68) | 0.63 (-0.09, 1.36) | 0.02 (-0.25, 0.29) | -0.24 (-0.80, 0.32) | -0.06 (-0.34, 0.21) | 0.09 (-0.35, 0.53) | 0.08 (-0.28, 0.44) |
| 2 | 1.14 (0.56, 1.73) | 0.04 (-0.33, 0.42) | 0.47 (-0.26, 1.21) | -0.05 (-0.32, 0.23) | -0.32 (-0.88, 0.25) | -0.08 (-0.36, 0.21) | -0.04 (-0.48, 0.41) | -0.02 (-0.38, 0.35) |
| 3 | 1.14 (0.55, 1.73) | 0.06 (-0.32, 0.44) | 0.52 (-0.22, 1.26) | -0.03 (-0.31, 0.24) | -0.27 (-0.83, 0.30) | -0.06 (-0.34, 0.22) | -0.01 (-0.45, 0.44) | 0.01 (-0.36, 0.38) |
| 4 | 1.26 (0.64, 1.88) | 0.07 (-0.43, 0.57) | 0.76 (-0.18, 1.71) | -0.09 (-0.46, 0.28) | -0.47 (-1.16, 0.24) | -0.13 (-0.49, 0.23) | -0.07 (-0.70, 0.56) | -0.03 (-0.54, 0.49) |
| Exposure averaged over a static time frame 2000‒2015 | | | | | | | | |
| 1 | 0.43 (-0.31, 1.18) | 0.38 (-0.05, 0.82) | 0.42 (-0.46, 1.31) | 0.15 (-0.14, 0.44) | 0.21 (-0.39, 0.82) | 0.07 (-0.22, 0.37) | 0.15 (-0.32, 0.62) | 0.12 (-0.26, 0.51) |
| 2 | 0.01 (-0.74, 0.77) | 0.14 (-0.30, 0.59) | 0.25 (-0.65, 1.16) | 0.10 (-0.20, 0.39) | 0.24 (-0.37, 0.86) | 0.09 (-0.21, 0.39) | 0.08 (-0.40, 0.57) | 0.08 (-0.32, 0.48) |
| 3 | 0.00 (-0.75, 0.76) | 0.11 (-0.34, 0.56) | 0.24 (-0.67, 1.15) | 0.08 (-0.22, 0.38) | 0.24 (-0.38, 0.86) | 0.08 (-0.22, 0.38) | 0.08 (-0.41, 0.56) | 0.07 (-0.33, 0.47) |
| 4 | 0.08 (-0.88, 1.05) | 0.20 (-0.40, 0.80) | 0.48 (-0.78, 1.75) | 0.15 (-0.27, 0.57) | 0.39 (-0.43, 1.22) | 0.12 (-0.28, 0.52) | 0.14 (-0.54, 0.82) | 0.12 (-0.43, 0.68) |

**Table S6.** Associations between air pollutants and percentage difference (95% confidence intervals) in creatinine-based and cystatin C-based eGFR, per interquartile range (IQR) higher exposure.

|  | **Percent difference in eGFR** | |
| --- | --- | --- |
|  | Creatinine-based eGFR | Cystatin C-based eGFR |
| PM_2.5_ (total) | 1.44 (0.75, 2.12) | 0.96 (0.12, 1.82) |
| PM_2.5_ (local) | 0.07 (-0.33, 0.48) | 0.22 (-0.28, 0.73) |
| PM_10_ (total) | 0.82 (-0.10, 1.75) | 0.80 (-0.35, 1.96) |
| PM_10_ (local) | 0.01 (-0.26, 0.28) | 0.06 (-0.27, 0.40) |
| PM_2.5–10_ (total) | -0.12 (-0.78, 0.54) | 0.17 (-0.65, 0.99) |
| PM_2.5–10_ (local) | -0.01 (-0.28, 0.26) | 0.02 (-0.32, 0.36) |
| NO_x_ (total) | 0.20 (-0.26, 0.67) | -0.01 (-0.59, 0.57) |
| NO_x_ (local) | 0.14 (-0.23, 0.52) | -0.03 (-0.49, 0.44) |

**Table S7.** Associations between air pollutants and percentage difference (95% confidence intervals) in eGFR per exposure quarter, with p-values for linear trend between exposure quarters.

|  | **Q2 vs. Q1** | **Q3 vs. Q1** | **Q4 vs. Q1** | **p** |
| --- | --- | --- | --- | --- |
| PM_2.5_ (total) | 0.26 (-0.4, 0.92) | 1.58 (0.62, 2.55) | 1.82 (0.60, 3.06) | 0.001 |
| PM_2.5_ (local) | -0.09 (-0.66, 0.48) | 0.14 (-0.56, 0.83) | 0.24 (-0.52, 1.01) | 0.474 |
| PM_10_ (total) | -0.22 (-0.88, 0.44) | 0.89 (-0.01, 1.80) | 0.85 (-0.27, 1.98) | 0.193 |
| PM_10_ (local) | -0.03 (-0.6, 0.54) | 0.01 (-0.60, 0.62) | 0.29 (-0.37, 0.94) | 0.400 |
| PM_2.5–10_ (total) | -0.18 (-0.77, 0.41) | 0.64 (-0.23, 1.52) | -0.27 (-1.46, 0.93) | 0.980 |
| PM_2.5–10_ (local) | 0.00 (-0.56, 0.56) | -0.17 (-0.76, 0.43) | 0.25 (-0.43, 0.93) | 0.656 |
| NO_x_ (total) | 0.08 (-0.53, 0.70) | 0.21 (-0.56, 0.99) | 0.54 (-0.33, 1.42) | 0.203 |
| NO_x_ (local) | 0.03 (-0.55, 0.63) | 0.32 (-0.37, 1.01) | 0.48 (-0.29, 1.25) | 0.169 |

**Table S8.** Associations between air pollutants and percentage difference (95% confidence intervals) in serum levels of included biomarkers, in the main model (model 2).

| **Biomarker** | **PM_2.5_ (total)** | **PM_2.5_ (local)** | **PM_10_ (total)** | **PM_10_ (local)** | **PM_2.5–10_ (total)** | **PM_2.5–10_ (local)** | **NO_x_ (total)** | **NO_x_ (local)** |
| --- | --- | --- | --- | --- | --- | --- | --- | --- |
| KIM-1 | -7.26 (-16.52, 3.02) | -4.88 (-9.11, -0.46) | -8.27 (-17.48, 1.98) | -2.55 (-5.43, 0.42) | -5.55 (-12.29, 1.71) | -2.06 (-5.01, 0.99) | -3.33 (-8.20, 1.78) | -2.74 (-6.71, 1.41) |
| MCP 1 | -0.58 (-6.44, 5.64) | -0.01 (-2.59, 2.65) | -0.70 (-6.59, 5.55) | -0.15 (-1.86, 1.60) | -0.48 (-4.63, 3.86) | -0.20 (-1.95, 1.58) | 0.04 (-2.89, 3.06) | 0.06 (-2.32, 2.50) |
| IL-6 | -0.52 (-10.67, 10.79) | -2.09 (-6.55, 2.57) | 0.92 (-9.46, 12.48) | -0.18 (-3.21, 2.93) | 1.06 (-6.32, 9.03) | 0.39 (-2.71, 3.59) | -1.75 (-6.80, 3.59) | -1.53 (-5.66, 2.77) |
| IL-18 | -3.76 (-11.08, 4.17) | -1.75 (-5.06, 1.66) | -0.89 (-8.48, 7.33) | -0.02 (-2.25, 2.27) | 0.46 (-4.99, 6.22) | 0.52 (-1.78, 2.86) | -1.79 (-5.53, 2.10) | -1.41 (-4.46, 1.74) |
| FGF-23 | -4.66 (-11.01, 2.13) | -2.16 (-5.03, 0.79) | -4.97 (-11.34, 1.85) | -1.24 (-3.16, 0.73) | -3.21 (-7.79, 1.60) | -1.06 (-3.03, 0.94) | -3.12 (-6.33, 0.21) | -2.52 (-5.15, 0.18) |
| MMP-2 | 7.21 (1.94, 12.75) | 1.86 (-0.33, 4.10) | 3.85 (-1.29, 9.25) | 0.97 (-0.47, 2.43) | 1.24 (-2.28, 4.89) | 0.79 (-0.68, 2.27) | 2.06 (-0.43, 4.61) | 1.58 (-0.43, 3.63) |
| MMP-7 | 0.50 (-4.90, 6.21) | 0.55 (-1.82, 2.98) | 1.62 (-3.88, 7.43) | 0.70 (-0.87, 2.30) | 1.38 (-2.49, 5.40) | 0.81 (-0.80, 2.45) | 1.16 (-1.55, 3.94) | 0.97 (-1.22, 3.21) |
| MMP-9 | -0.44 (-10.90, 11.24) | -0.41 (-5.07, 4.48) | -2.91 (-13.16, 8.56) | -0.82 (-3.90, 2.37) | -2.65 (-9.95, 5.25) | -1.01 (-4.15, 2.23) | -2.78 (-7.93, 2.65) | -2.29 (-6.49, 2.11) |
| Uric acid | 1.28 (-0.70, 3.30) | 0.55 (-0.52, 1.64) | 2.31 (-0.21, 4.89) | 0.66 (-0.06, 1.40) | 1.56 (-0.22, 3.38) | 0.75 (0.01, 1.50) | 0.50 (-0.83, 1.84) | 0.41 (-0.67, 1.49) |

**Table S9.** Associations between air pollutants and percentage difference (95% confidence intervals) in serum levels of included biomarkers per exposure quarter, with p-values for linear trend between exposure quarters.

| **Exposure** | **Biomarker** | **Q2 vs. Q1** | **Q3 vs. Q1** | **Q4 vs. Q1** | **p** |
| --- | --- | --- | --- | --- | --- |
| PM_2.5_ (total) | KIM-1 | -2.04 (-8.79, 5.22) | 7.29 (-14.87, 35.23) | -2.32 (-23.43, 24.60) | 0.076 |
|  | MCP 1 | -1.66 (-5.59, 2.43) | 12.66 (-1.19, 28.45) | 11.83 (-2.60, 28.39) | 0.855 |
|  | IL-6 | -3.39 (-10.32, 4.08) | 6.31 (-16.47, 35.30) | 3.31 (-19.84, 33.15) | 0.375 |
|  | IL-18 | 3.75 (-1.67, 9.46) | -6.38 (-21.33, 11.39) | -5.66 (-21.43, 13.27) | 0.429 |
|  | FGF-23 | 1.22 (-3.41, 6.06) | 1.00 (-13.20, 17.52) | -1.78 (-16.26, 15.19) | 0.710 |
|  | MMP-2 | 3.64 (0.19, 7.20) | 15.69 (3.78, 28.98) | 16.02 (3.47, 30.10) | 0.028 |
|  | MMP-7 | 0.94 (-2.75, 4.76) | 14.66 (1.64, 29.34) | 12.63 (-0.78, 27.86) | 0.693 |
|  | MMP-9 | -6.68 (-13.41, 0.58) | 0.60 (-20.93, 27.98) | -1.18 (-23.31, 27.35) | 0.160 |
|  | Uric acid | 0.20 (-6.56, 7.46) | -0.71 (-7.41, 6.46) | 0.43 (-6.48, 7.84) | 0.854 |
| PM_2.5_ (local) | KIM-1 | -3.60 (-8.94, 2.05) | -4.46 (-10.91, 2.45) | -8.32 (-15.34, -0.71) | 0.038 |
|  | MCP 1 | -0.79 (-3.96, 2.48) | 0.78 (-3.17, 4.88) | -1.60 (-5.99, 2.99) | 0.593 |
|  | IL-6 | -4.30 (-9.81, 1.55) | -3.58 (-10.34, 3.70) | -10.14 (-17.29, -2.36) | 0.018 |
|  | IL-18 | 1.68 (-2.57, 6.13) | 0.96 (-4.20, 6.40) | -1.27 (-7.01, 4.82) | 0.692 |
|  | FGF-23 | 0.85 (-2.84, 4.68) | 0.59 (-3.90, 5.30) | -0.97 (-6.01, 4.34) | 0.736 |
|  | MMP-2 | 1.25 (-1.44, 4.01) | 1.31 (-1.99, 4.72) | 3.95 (0.09, 7.96) | 0.057 |
|  | MMP-7 | 0.80 (-2.14, 3.84) | 1.84 (-1.80, 5.61) | 1.20 (-2.91, 5.50) | 0.524 |
|  | MMP-9 | -4.30 (-9.83, 1.58) | -7.37 (-13.92, -0.33) | -6.48 (-13.99, 1.69) | 0.097 |
|  | Uric acid | 0.54 (-1.71, 2.85) | -0.92 (-3.22, 1.44) | 0.71 (-1.68, 3.17) | 0.585 |
| PM_10_ (total) | KIM-1 | -2.81 (-8.97, 3.76) | -3.67 (-12.26, 5.75) | -5.56 (-16.96, 7.41) | 0.342 |
|  | MCP 1 | 1.39 (-2.32, 5.24) | -0.29 (-5.46, 5.16) | 1.19 (-5.99, 8.92) | 0.770 |
|  | IL-6 | -3.40 (-9.77, 3.42) | -8.12 (-16.64, 1.26) | -9.02 (-20.43, 4.03) | 0.113 |
|  | IL-18 | 0.28 (-4.53, 5.34) | -3.55 (-10.09, 3.45) | -2.71 (-11.67, 7.16) | 0.512 |
|  | FGF-23 | 2.17 (-2.11, 6.65) | -2.61 (-8.38, 3.53) | -2.39 (-10.27, 6.19) | 0.634 |
|  | MMP-2 | 3.11 (-0.03, 6.34) | 3.98 (-0.50, 8.67) | 4.69 (-1.51, 11.28) | 0.070 |
|  | MMP-7 | 3.84 (0.36, 7.44) | 1.46 (-3.35, 6.52) | 0.93 (-5.61, 7.92) | 0.490 |
|  | MMP-9 | -2.81 (-9.23, 4.07) | -4.41 (-13.30, 5.40) | 0.86 (-11.89, 15.44) | 0.749 |
|  | Uric acid | 0.93 (-3.65, 5.74) | -2.87 (-11.85, 7.02) | -2.09 (-11.21, 7.96) | 0.330 |
| PM_10_ (local) | KIM-1 | -5.46 (-10.72, 0.12) | -4.04 (-9.78, 2.07) | -5.68 (-11.74, 0.80) | 0.137 |
|  | MCP 1 | -0.16 (-3.36, 3.15) | 0.15 (-3.31, 3.74) | -1.67 (-5.33, 2.13) | 0.453 |
|  | IL-6 | -4.19 (-9.74, 1.71) | -8.43 (-14.13, -2.35) | -5.41 (-11.73, 1.35) | 0.058 |
|  | IL-18 | 0.80 (-3.45, 5.24) | -2.10 (-6.54, 2.54) | -0.70 (-5.53, 4.38) | 0.537 |
|  | FGF-23 | -1.30 (-4.93, 2.48) | -1.24 (-5.15, 2.83) | -3.22 (-7.33, 1.08) | 0.172 |
|  | MMP-2 | -0.54 (-3.19, 2.19) | 1.92 (-1.01, 4.94) | 0.18 (-2.92, 3.38) | 0.555 |
|  | MMP-7 | 0.48 (-2.48, 3.52) | 2.19 (-1.04, 5.53) | 1.04 (-2.39, 4.59) | 0.393 |
|  | MMP-9 | -3.38 (-8.99, 2.59) | -5.83 (-11.72, 0.45) | -3.18 (-9.69, 3.79) | 0.271 |
|  | Uric acid | -0.42 (-2.53, 1.74) | 0.11 (-1.99, 2.26) | -0.06 (-2.12, 2.05) | 0.907 |
| PM_2.5‒10_ (total) | KIM-1 | -1.31 (-6.76, 4.46) | -2.76 (-10.98, 6.22) | -0.69 (-12.98, 13.34) | 0.655 |
|  | MCP 1 | -0.64 (-3.79, 2.62) | -0.68 (-5.59, 4.48) | -2.14 (-9.28, 5.56) | 0.601 |
|  | IL-6 | -5.84 (-11.25, -0.11) | -3.41 (-11.89, 5.90) | 2.48 (-10.69, 17.59) | 0.432 |
|  | IL-18 | -1.84 (-5.94, 2.44) | -1.96 (-8.26, 4.77) | -1.02 (-10.37, 9.31) | 0.535 |
|  | FGF-23 | -0.32 (-3.96, 3.45) | -2.28 (-7.77, 3.54) | -6.46 (-14.21, 1.99) | 0.257 |
|  | MMP-2 | 2.23 (-0.46, 5.00) | 1.57 (-2.60, 5.93) | -0.76 (-6.80, 5.67) | 0.469 |
|  | MMP-7 | 3.89 (0.86, 7.00) | -0.86 (-5.31, 3.80) | -0.26 (-6.89, 6.84) | 0.496 |
|  | MMP-9 | -9.29 (-14.50, -3.77) | -0.52 (-9.34, 9.16) | 0.38 (-12.63, 15.32) | 0.249 |
|  | Uric acid | 1.55 (-1.88, 5.09) | 2.13 (-3.38, 7.95) | 0.00 (-5.69, 6.05) | 0.467 |
| PM_2.5‒10_ (local) | KIM-1 | -4.56 (-9.81, 1.01) | -5.13 (-10.72, 0.82) | -6.58 (-12.76, 0.05) | 0.054 |
|  | MCP 1 | 0.85 (-2.35, 4.16) | -0.18 (-3.58, 3.34) | 0.18 (-3.67, 4.18) | 0.952 |
|  | IL-6 | -2.28 (-7.88, 3.66) | -6.71 (-12.43, -0.62) | -2.55 (-9.26, 4.66) | 0.243 |
|  | IL-18 | 0.23 (-3.95, 4.58) | -3.73 (-8.02, 0.77) | 0.06 (-4.96, 5.35) | 0.594 |
|  | FGF-23 | -0.31 (-3.93, 3.46) | -2.62 (-6.42, 1.33) | -0.55 (-4.91, 4.01) | 0.545 |
|  | MMP-2 | 1.37 (-1.31, 4.12) | 1.24 (-1.63, 4.18) | 1.27 (-1.97, 4.61) | 0.460 |
|  | MMP-7 | 3.16 (0.16, 6.25) | 3.84 (0.61, 7.18) | 2.19 (-1.39, 5.90) | 0.167 |
|  | MMP-9 | -6.74 (-12.10, -1.05) | -7.60 (-13.29, -1.55) | -3.02 (-9.74, 4.20) | 0.285 |
|  | Uric acid | 1.11 (-0.88, 3.15) | -0.42 (-2.50, 1.70) | 1.27 (-0.89, 3.49) | 0.378 |
| NO_x_ (total) | KIM-1 | -2.74 (-8.52, 3.41) | -2.52 (-10.14, 5.75) | -5.00 (-13.48, 4.32) | 0.302 |
|  | MCP 1 | 0.99 (-2.47, 4.59) | 2.07 (-2.56, 6.93) | -0.40 (-5.58, 5.07) | 0.897 |
|  | IL-6 | -3.38 (-9.35, 2.99) | -4.32 (-12.10, 4.15) | -6.02 (-14.74, 3.61) | 0.223 |
|  | IL-18 | 3.04 (-1.59, 7.90) | 2.19 (-3.87, 8.63) | 0.45 (-6.37, 7.76) | 0.955 |
|  | FGF-23 | 2.52 (-1.51, 6.72) | 0.31 (-4.90, 5.79) | -0.61 (-6.51, 5.66) | 0.766 |
|  | MMP-2 | 2.05 (-0.86, 5.05) | 4.33 (0.39, 8.43) | 4.25 (-0.26, 8.97) | 0.062 |
|  | MMP-7 | -0.20 (-3.33, 3.04) | 4.81 (0.47, 9.35) | 1.68 (-3.16, 6.75) | 0.410 |
|  | MMP-9 | -1.70 (-7.81, 4.81) | -4.61 (-12.40, 3.88) | -5.22 (-14.08, 4.55) | 0.273 |
|  | Uric acid | 0.22 (-3.20, 3.77) | -0.40 (-4.19, 3.55) | 0.06 (-3.80, 4.08) | 0.920 |
| NO_x_ (local) | KIM-1 | -3.28 (-8.78, 2.54) | -3.10 (-9.79, 4.09) | -5.34 (-12.71, 2.64) | 0.222 |
|  | MCP 1 | 0.79 (-2.51, 4.20) | -0.18 (-4.19, 3.99) | -0.40 (-4.91, 4.33) | 0.783 |
|  | IL-6 | -4.66 (-10.29, 1.33) | -3.90 (-10.80, 3.54) | -7.31 (-14.80, 0.85) | 0.112 |
|  | IL-18 | 1.32 (-3.03, 5.87) | -1.36 (-6.52, 4.09) | -1.91 (-7.70, 4.24) | 0.411 |
|  | FGF-23 | 2.42 (-1.43, 6.41) | 0.80 (-3.81, 5.63) | -1.14 (-6.24, 4.24) | 0.546 |
|  | MMP-2 | 0.97 (-1.78, 3.80) | 1.94 (-1.47, 5.46) | 2.36 (-1.49, 6.37) | 0.220 |
|  | MMP-7 | -0.13 (-3.13, 2.96) | 3.93 (0.13, 7.87) | 1.66 (-2.53, 6.04) | 0.250 |
|  | MMP-9 | -2.73 (-8.49, 3.40) | -5.54 (-12.38, 1.84) | -6.47 (-14.09, 1.83) | 0.111 |
|  | Uric acid | -1.18 (-3.60, 1.31) | -0.84 (-3.48, 1.88) | -0.83 (-3.51, 1.93) | 0.886 |

**Table S10.** Associations between air pollutants and percentage difference (95% confidence intervals) in serum levels of included biomarkers, in the main model (model 2).

| **Site** | **PM_2.5_ (total)** | **PM_2.5_ (local)** | **PM_10_ (total)** | **PM_10_ (local)** | **PM_2.5–10_ (total)** | **PM_2.5–10_ (local)** | **NO_x_ (total)** | **NO_x_ (local)** |
| --- | --- | --- | --- | --- | --- | --- | --- | --- |
| KIM-1 |  |  |  |  |  |  |  |  |
| Umeå | -10.09 (-31.53, 18.06) | -7.31 (-18.05, 4.83) | -9.47 (-28.32, 14.33) | -3.37 (-9.70, 3.41) | -6.69 (-20.76, 9.86) | -2.99 (-9.35, 3.80) | -3.36 (-20.58, 17.60) | -2.83 (-17.22, 14.06) |
| Uppsala | -5.52 (-30.24, 27.96) | -4.60 (-18.64, 11.86) | -12.40 (-40.07, 28.05) | -4.71 (-14.86, 6.64) | -11.63 (-34.49, 19.22) | -5.89 (-16.97, 6.68) | -5.42 (-19.32, 10.87) | -4.42 (-15.97, 8.72) |
| Stockholm | -12.60 (-34.95, 17.43) | -10.79 (-20.99, 0.73) | -12.68 (-36.80, 20.65) | -4.50 (-12.74, 4.51) | -7.92 (-27.30, 16.62) | -2.35 (-11.55, 7.82) | -1.52 (-11.66, 9.78) | -1.35 (-9.64, 7.71) |
| Linköping | 1.73 (-31.79, 51.73) | -4.11 (-19.65, 14.44) | -1.34 (-24.82, 29.47) | -1.16 (-8.16, 6.38) | -1.23 (-16.54, 16.90) | -0.88 (-7.28, 5.96) | -5.70 (-25.28, 19.00) | -4.89 (-21.16, 14.73) |
| Gothenburg | -3.64 (-19.82, 15.81) | -2.25 (-9.24, 5.27) | -4.50 (-20.19, 14.27) | -1.00 (-5.88, 4.13) | -3.05 (-14.25, 9.62) | -0.67 (-5.66, 4.59) | -3.33 (-11.60, 5.71) | -2.80 (-9.60, 4.50) |
| Malmö | -10.42 (-31.68, 17.45) | -3.79 (-14.78, 8.63) | -20.10 (-47.74, 22.15) | -4.99 (-16.32, 7.87) | -20.69 (-47.14, 19.01) | -7.26 (-22.04, 10.32) | -8.05 (-19.25, 4.71) | -6.14 (-15.47, 4.22) |
| *Total* | *-7.13 (-17.94, 3.68)* | *-4.99 (-9.67, -0.31)* | *-8.12 (-18.95, 2.71)* | *-2.48 (-5.55, 0.59)* | *-5.37 (-12.93, 2.20)* | *-1.96 (-5.09, 1.17)* | *-4.16 (-9.47, 1.15)* | *-3.38 (-7.68, 0.92)* |
| MCP-1 |  |  |  |  |  |  |  |  |
| Umeå | -1.95 (-17.06, 15.91) | -0.30 (-7.58, 7.56) | -3.93 (-16.75, 10.86) | -0.92 (-4.96, 3.29) | -3.31 (-12.53, 6.87) | -1.17 (-5.19, 3.02) | -2.12 (-13.23, 10.41) | -1.58 (-10.80, 8.59) |
| Uppsala | -2.85 (-20.68, 18.98) | 2.65 (-7.70, 14.15) | 4.42 (-18.97, 34.56) | 2.36 (-5.04, 10.33) | 7.47 (-11.95, 31.18) | 2.83 (-5.40, 11.77) | 2.76 (-7.56, 14.23) | 2.33 (-6.08, 11.49) |
| Stockholm | -5.75 (-19.76, 10.71) | -5.25 (-11.30, 1.20) | -6.80 (-21.85, 11.14) | -2.76 (-7.41, 2.12) | -4.67 (-16.18, 8.43) | -2.10 (-7.24, 3.32) | -1.88 (-7.47, 4.05) | -1.61 (-6.17, 3.17) |
| Linköping | 7.01 (-15.14, 34.95) | 5.95 (-4.41, 17.43) | 6.86 (-8.73, 25.11) | 2.06 (-2.21, 6.51) | 4.26 (-5.44, 14.96) | 1.71 (-2.15, 5.73) | 8.01 (-5.76, 23.78) | 6.34 (-4.72, 18.70) |
| Gothenburg | -1.14 (-9.60, 8.11) | -0.37 (-3.90, 3.29) | -3.09 (-11.19, 5.75) | -0.89 (-3.30, 1.57) | -2.49 (-8.14, 3.50) | -1.13 (-3.57, 1.38) | -1.44 (-5.65, 2.95) | -1.16 (-4.59, 2.39) |
| Malmö | 7.21 (-7.84, 24.71) | 3.10 (-3.66, 10.34) | 9.89 (-13.38, 39.41) | 2.20 (-4.84, 9.75) | 6.28 (-15.46, 33.60) | 1.22 (-8.22, 11.64) | 1.83 (-5.34, 9.54) | 1.55 (-4.25, 7.72) |
| *Total* | *-0.29 (-6.19, 5.62)* | *-0.09 (-2.60, 2.43)* | *-1.17 (-7.07, 4.74)* | *-0.34 (-2.01, 1.33)* | *-1.02 (-5.12, 3.09)* | *-0.45 (-2.16, 1.25)* | *-0.38 (-3.25, 2.48)* | *-0.30 (-2.62, 2.02)* |
| IL-6 |  |  |  |  |  |  |  |  |
| Umeå | 11.89 (-14.47, 46.38) | 2.97 (-8.82, 16.28) | 3.25 (-17.99, 29.99) | 0.01 (-6.47, 6.93) | 0.27 (-14.66, 17.81) | -0.59 (-7.01, 6.29) | 0.43 (-17.25, 21.89) | 0.17 (-14.47, 17.33) |
| Uppsala | 24.33 (-8.30, 68.58) | 6.83 (-8.95, 25.35) | 13.97 (-22.17, 66.92) | 1.71 (-9.18, 13.90) | 1.25 (-25.06, 36.82) | 0.06 (-11.79, 13.50) | 0.25 (-14.55, 17.61) | -0.05 (-12.19, 13.77) |
| Stockholm | -22.60 (-43.11, 5.29) | -12.45 (-22.86, -0.62) | -28.82 (-49.16, -0.33) | -9.54 (-17.64, -0.64) | -21.59 (-38.69, 0.28) | -9.78 (-18.62, 0.01) | -7.20 (-17.13, 3.94) | -5.88 (-14.12, 3.14) |
| Linköping | -15.38 (-42.22, 23.94) | -5.37 (-20.07, 12.03) | 3.77 (-19.96, 34.53) | 0.94 (-5.91, 8.28) | 4.98 (-10.62, 23.30) | 1.57 (-4.70, 8.25) | -3.47 (-22.70, 20.56) | -2.87 (-18.80, 16.19) |
| Gothenburg | -3.50 (-20.18, 16.66) | -2.31 (-9.51, 5.45) | 5.17 (-12.62, 26.58) | 1.80 (-3.37, 7.25) | 5.92 (-6.69, 20.23) | 3.31 (-2.05, 8.95) | -1.70 (-10.37, 7.80) | -1.43 (-8.53, 6.23) |
| Malmö | 15.38 (-14.93, 56.50) | 4.23 (-9.08, 19.48) | 25.58 (-22.13, 102.51) | 3.63 (-10.17, 19.56) | 20.46 (-23.72, 90.23) | 3.30 (-15.04, 25.60) | 7.81 (-6.86, 24.79) | 5.84 (-5.93, 19.09) |
| *Total* | *0.90 (-12.69, 14.49)* | *-1.66 (-6.99, 3.66)* | *1.67 (-10.19, 13.53)* | *-0.03 (-3.24, 3.18)* | *1.28 (-6.82, 9.39)* | *0.36 (-3.05, 3.77)* | *-1.47 (-6.98, 4.04)* | *-1.32 (-5.78, 3.15)* |
| IL-18 |  |  |  |  |  |  |  |  |
| Umeå | -16.31 (-31.37, 2.05) | -7.22 (-15.18, 1.49) | -13.92 (-27.38, 2.02) | -4.08 (-8.70, 0.78) | -9.52 (-19.67, 1.92) | -3.97 (-8.59, 0.89) | -10.13 (-22.11, 3.68) | -8.35 (-18.45, 2.99) |
| Uppsala | -0.66 (-20.86, 24.70) | 4.22 (-7.49, 17.43) | 8.68 (-18.23, 44.46) | 3.45 (-4.93, 12.56) | 11.23 (-11.12, 39.21) | 3.99 (-5.34, 14.23) | 5.30 (-6.53, 18.61) | 4.49 (-5.13, 15.08) |
| Stockholm | -10.85 (-28.67, 11.42) | -8.07 (-16.13, 0.76) | -15.10 (-33.48, 8.35) | -5.45 (-11.67, 1.20) | -11.39 (-25.86, 5.90) | -5.19 (-12.01, 2.17) | -8.62 (-15.80, -0.84) | -7.13 (-13.07, -0.78) |
| Linköping | 10.26 (-20.90, 53.70) | 5.62 (-8.81, 22.34) | 8.56 (-13.38, 36.06) | 1.94 (-4.10, 8.36) | 5.13 (-8.60, 20.92) | 1.62 (-3.86, 7.41) | 4.77 (-13.65, 27.12) | 3.80 (-11.19, 21.31) |
| Gothenburg | 6.30 (-6.99, 21.49) | 2.25 (-3.11, 7.91) | 10.19 (-3.27, 25.52) | 3.65 (-0.08, 7.52) | 7.30 (-1.85, 17.30) | 4.38 (0.55, 8.36) | 3.53 (-2.98, 10.48) | 3.02 (-2.26, 8.59) |
| Malmö | -6.61 (-23.43, 13.89) | -3.75 (-11.93, 5.20) | -5.97 (-31.12, 28.37) | -2.76 (-11.40, 6.72) | -0.37 (-26.02, 34.16) | -1.76 (-13.50, 11.57) | -2.44 (-11.31, 7.31) | -2.05 (-9.29, 5.76) |
| *Total* | *-3.00 (-11.28, 5.28)* | *-1.77 (-6.38, 2.83)* | *-1.23 (-12.03, 9.57)* | *-0.38 (-3.94, 3.19)* | *0.08 (-7.84, 8.00)* | *0.07 (-3.65, 3.80)* | *-1.65 (-7.25, 3.95)* | *-1.31 (-5.95, 3.34)* |
| FGF-23 |  |  |  |  |  |  |  |  |
| Umeå | 0.75 (-16.28, 21.24) | 0.13 (-7.91, 8.88) | -1.87 (-16.27, 15.00) | -0.66 (-5.14, 4.02) | -1.99 (-12.29, 9.52) | -0.91 (-5.37, 3.76) | -2.44 (-14.62, 11.48) | -2.05 (-12.16, 9.21) |
| Uppsala | 0.34 (-16.46, 20.53) | 3.49 (-6.00, 13.93) | 4.23 (-17.14, 31.11) | 2.91 (-3.86, 10.15) | 4.96 (-12.41, 25.78) | 3.39 (-4.15, 11.53) | 3.43 (-6.04, 13.85) | 2.88 (-4.83, 11.21) |
| Stockholm | -15.81 (-30.43, 1.88) | -8.78 (-15.67, -1.33) | -23.59 (-37.96, -5.89) | -7.36 (-12.59, -1.81) | -18.63 (-30.12, -5.26) | -7.93 (-13.62, -1.87) | -7.30 (-13.58, -0.57) | -5.97 (-11.15, -0.49) |
| Linköping | -12.14 (-32.17, 13.82) | -8.96 (-18.80, 2.07) | -5.54 (-20.79, 12.65) | -1.50 (-6.08, 3.31) | -2.67 (-12.73, 8.55) | -0.77 (-4.97, 3.62) | -5.91 (-19.07, 9.40) | -4.95 (-15.83, 7.33) |
| Gothenburg | -2.90 (-15.39, 11.44) | -1.37 (-6.69, 4.27) | -3.02 (-15.22, 10.93) | -0.88 (-4.57, 2.94) | -1.90 (-10.52, 7.56) | -0.79 (-4.55, 3.12) | -2.36 (-8.68, 4.40) | -2.00 (-7.18, 3.47) |
| Malmö | -0.17 (-14.30, 16.30) | 0.74 (-5.92, 7.87) | -6.30 (-26.24, 19.03) | -0.79 (-7.65, 6.57) | -10.81 (-29.04, 12.10) | -2.97 (-12.01, 7.01) | -1.00 (-7.99, 6.53) | -0.52 (-6.23, 5.53) |
| *Total* | *-4.04 (-11.16, 3.07)* | *-2.18 (-5.97, 1.61)* | *-5.82 (-13.19, 1.55)* | *-1.49 (-3.84, 0.86)* | *-4.49 (-10.46, 1.48)* | *-1.51 (-3.98, 0.97)* | *-2.80 (-6.29, 0.68)* | *-2.23 (-5.05, 0.58)* |
| MMP-2 |  |  |  |  |  |  |  |  |
| Umeå | 8.61 (-5.99, 25.47) | 1.78 (-4.67, 8.66) | 6.27 (-6.08, 20.24) | 1.38 (-2.20, 5.09) | 3.87 (-4.73, 13.25) | 1.48 (-2.09, 5.19) | 4.68 (-5.66, 16.14) | 3.62 (-4.81, 12.80) |
| Uppsala | 8.80 (-6.46, 26.56) | 3.78 (-4.13, 12.34) | 11.13 (-8.02, 34.26) | 3.03 (-2.58, 8.96) | 7.42 (-7.43, 24.65) | 3.47 (-2.77, 10.11) | 3.29 (-4.55, 11.77) | 2.59 (-3.77, 9.37) |
| Stockholm | 17.44 (2.83, 34.13) | 4.62 (-0.94, 10.49) | 8.79 (-5.97, 25.86) | 1.92 (-2.14, 6.14) | 1.73 (-8.57, 13.18) | 1.06 (-3.35, 5.68) | 2.69 (-2.18, 7.80) | 2.10 (-1.83, 6.18) |
| Linköping | 12.76 (-5.98, 35.24) | 2.39 (-5.56, 11.00) | 7.67 (-4.85, 21.84) | 1.90 (-1.46, 5.36) | 4.19 (-3.50, 12.48) | 1.87 (-1.18, 5.01) | 3.67 (-6.85, 15.38) | 2.88 (-5.62, 12.15) |
| Gothenburg | 0.26 (-7.97, 9.23) | -0.18 (-3.57, 3.32) | -2.31 (-10.15, 6.21) | -0.37 (-2.68, 2.01) | -2.21 (-7.64, 3.54) | -0.45 (-2.81, 1.97) | 0.29 (-3.82, 4.56) | 0.21 (-3.13, 3.66) |
| Malmö | 8.39 (-4.41, 22.90) | 3.25 (-2.41, 9.24) | 7.44 (-11.84, 30.93) | 1.88 (-3.98, 8.11) | 0.24 (-17.13, 21.24) | 0.25 (-7.59, 8.75) | 2.37 (-3.65, 8.78) | 1.69 (-3.17, 6.79) |
| *Total* | *6.84 (1.70, 11.97)* | *1.83 (-0.38, 4.05)* | *3.80 (-1.36, 8.96)* | *1.01 (-0.45, 2.47)* | *1.30 (-2.29, 4.90)* | *0.85 (-0.64, 2.33)* | *1.98 (-0.51, 4.47)* | *1.52 (-0.50, 3.53)* |
| MMP-7 |  |  |  |  |  |  |  |  |
| Umeå | 8.63 (-4.84, 24.02) | 5.23 (-0.88, 11.72) | 8.22 (-3.39, 21.22) | 2.87 (-0.46, 6.32) | 5.70 (-2.37, 14.44) | 2.79 (-0.53, 6.23) | 9.96 (-0.03, 20.95) | 8.05 (-0.04, 16.79) |
| Uppsala | 17.46 (0.41, 37.40) | 9.72 (1.06, 19.13) | 24.52 (2.34, 51.51) | 6.03 (0.04, 12.39) | 17.28 (0.46, 36.92) | 6.05 (-0.61, 13.16) | 7.04 (-1.41, 16.22) | 5.58 (-1.23, 12.86) |
| Stockholm | -13.25 (-28.09, 4.67) | -7.88 (-14.73, -0.49) | -7.06 (-24.34, 14.17) | -2.49 (-7.93, 3.27) | -1.36 (-15.13, 14.65) | -0.43 (-6.51, 6.05) | -4.17 (-10.56, 2.69) | -3.41 (-8.65, 2.14) |
| Linköping | 3.95 (-16.36, 29.20) | 3.08 (-6.37, 13.49) | 0.25 (-13.53, 16.23) | 0.23 (-3.70, 4.32) | -0.29 (-9.02, 9.28) | -0.04 (-3.60, 3.66) | 4.81 (-7.65, 18.94) | 3.91 (-6.16, 15.08) |
| Gothenburg | -0.61 (-9.13, 8.72) | -0.42 (-3.95, 3.25) | -2.38 (-10.57, 6.55) | -0.58 (-3.00, 1.90) | -2.01 (-7.71, 4.04) | -0.67 (-3.14, 1.86) | 0.37 (-3.91, 4.85) | 0.24 (-3.24, 3.85) |
| Malmö | -0.35 (-12.11, 12.98) | 3.50 (-2.16, 9.48) | 1.38 (-16.73, 23.43) | 3.38 (-2.52, 9.64) | 3.06 (-14.61, 24.40) | 3.68 (-4.33, 12.36) | 1.96 (-4.00, 8.29) | 2.09 (-2.75, 7.16) |
| *Total* | *2.36 (-4.59, 9.31)* | *1.88 (-2.21, 5.98)* | *2.81 (-4.03, 9.64)* | *1.17 (-0.91, 3.25)* | *1.83 (-2.64, 6.30)* | *0.99 (-0.83, 2.81)* | *2.09 (-1.49, 5.68)* | *1.77 (-1.15, 4.69)* |
| MMP-9 |  |  |  |  |  |  |  |  |
| Umeå | -11.38 (-35.27, 21.33) | -4.96 (-17.57, 9.58) | -11.71 (-32.52, 15.53) | -3.30 (-10.58, 4.56) | -8.59 (-24.26, 10.33) | -3.40 (-10.65, 4.44) | -8.52 (-27.03, 14.70) | -7.01 (-22.69, 11.85) |
| Uppsala | -20.27 (-41.41, 8.48) | -17.66 (-29.90, -3.27) | -35.48 (-56.08, -5.22) | -12.83 (-22.19, -2.33) | -31.66 (-49.48, -7.55) | -13.70 (-23.95, -2.06) | -16.51 (-28.89, -1.98) | -13.62 (-24.15, -1.62) |
| Stockholm | -17.69 (-39.58, 12.12) | -7.15 (-18.21, 5.40) | -21.46 (-43.99, 10.13) | -5.95 (-14.40, 3.34) | -15.42 (-33.95, 8.30) | -6.40 (-15.61, 3.81) | -6.24 (-16.24, 4.94) | -5.04 (-13.30, 4.02) |
| Linköping | 6.21 (-30.52, 62.35) | 5.60 (-12.53, 27.49) | 17.08 (-12.25, 56.21) | 4.40 (-3.44, 12.87) | 11.82 (-6.47, 33.68) | 4.32 (-2.81, 11.98) | 16.00 (-9.60, 48.86) | 12.90 (-7.66, 38.04) |
| Gothenburg | 8.67 (-10.04, 31.28) | 3.39 (-4.19, 11.58) | 3.44 (-13.99, 24.40) | 1.18 (-3.94, 6.58) | 0.54 (-11.37, 14.05) | 0.56 (-4.63, 6.03) | 2.87 (-6.19, 12.80) | 2.35 (-5.01, 10.29) |
| Malmö | 29.19 (-1.52, 69.48) | 12.00 (-0.83, 26.50) | 33.73 (-12.78, 105.05) | 6.68 (-6.16, 21.27) | 12.90 (-25.17, 70.35) | 0.48 (-15.74, 19.83) | 3.09 (-9.59, 17.54) | 2.11 (-8.15, 13.51) |
| *Total* | *-1.01 (-16.31, 14.29)* | *-1.30 (-9.38, 6.78)* | *-4.74 (-22.46, 12.98)* | *-1.47 (-6.39, 3.45)* | *-5.30 (-17.72, 7.11)* | *-2.05 (-6.75, 2.66)* | *-2.45 (-10.00, 5.10)* | *-2.02 (-8.11, 4.08)* |
| Uric acid |  |  |  |  |  |  |  |  |
| Umeå | 3.62 (-3.83, 11.65) | 1.07 (-2.35, 4.61) | 2.19 (-4.00, 8.78) | 0.42 (-1.39, 2.26) | 1.19 (-3.05, 5.61) | 0.34 (-1.42, 2.14) | 1.32 (-4.43, 7.41) | 1.01 (-3.70, 5.95) |
| Gothenburg | 0.84 (-1.34, 3.07) | 0.85 (-0.36, 2.09) | 2.40 (-0.43, 5.31) | 0.89 (0.07, 1.71) | 1.84 (-0.14, 3.86) | 0.96 (0.14, 1.79) | 0.79 (-0.70, 2.30) | 0.70 (-0.50, 1.92) |
| Malmö | 3.90 (-1.65, 9.76) | -0.46 (-3.32, 2.50) | 2.99 (-6.18, 13.07) | -0.86 (-3.73, 2.09) | -1.57 (-9.62, 7.20) | -1.41 (-5.09, 2.40) | 0.54 (-2.89, 4.08) | 0.21 (-2.54, 3.04) |
| *Total* | *1.41 (-0.55, 3.36)* | *0.70 (-0.37, 1.76)* | *2.38 (-0.09, 4.84)* | *0.71 (-0.01, 1.42)* | *1.57 (-0.18, 3.32)* | *0.77 (0.03, 1.50)* | *0.77 (-0.55, 2.10)* | *0.64 (-0.43, 1.72)* |
